# Covid-19 and chronic conditions: lessons to strengthen care in primary health care

**DOI:** 10.11606/s1518-8787.2026060006917

**Published:** 2026-05-01

**Authors:** Marina Souza Vieira, Thais Regis Aranha Rossi, Carla Maria Lima Santos, Laio Magno, Ines Dourado

**Affiliations:** I Universidade do Estado da Bahia. Departamento de Ciências da Vida. Salvador, BA, Brasil; II Secretaria de Saúde do Estado da Bahia. Salvador, BA, Brasil; III Universidade Federal da Bahia. Instituto de Saúde Coletiva. Salvador, BA, Brasil; IV Universidade Estadual de Feira de Santana. Departamento de Saúde. Feira de Santana, BA, Brasil; V Fundação Oswaldo Cruz. Instituto Gonçalo Moniz. Salvador, BA, Brasil

**Keywords:** Chronic Care Model, Covid-19, Pandemic Preparedness, Primary Health Care

## Abstract

**OBJECTIVE:**

To analyze the work process of Primary Health Care (PHC) professionals regarding care practices aimed at users with hypertension and diabetes during the Covid-19 pandemic in a capital in Northeastern Brazil.

**METHODS:**

Thirty-five semi-structured interviews were conducted with professionals who provided care and followed up this subpopulation in PHC. In addition, municipal documents and news reports from the Municipal Department of Health about Covid-19 and PHC were analyzed. The study criteria were Primary Health Care workers who acted in the Covid-19 pandemic in the same Family Health unit. Data were analyzed according to the health work process reference of Mendes-Gonçalves.

**RESULTS:**

The pandemic has imposed significant changes in PHC practices. We observed priority of spontaneous demand, weaknesses in continuous monitoring of users, emphasis on prescriptions renewal, and work in vast territories, with unattended areas, by the Family Health Strategy.

**CONCLUSIONS:**

Health practices aimed at users with chronic conditions, such as hypertension and diabetes, remain fragmented and should be resumed, but based on equity, with planning, monitoring, and knowledge of their territory in such a way to provide comprehensive and problem-solving access to the population that remains "isolated," even with the end of the pandemic.

## INTRODUCTION

The Brazilian Unified Health System played an important role in the management and control of Covid-19^1^. However, the period was marked by manifestations of denialism regarding the severity of the health crisis, in addition to technical differences in the conduct of the Federal Government, which resulted in political and social polarizations amid the greatest public health emergency of the 21st century^2,3^.

The Family Health Strategy (FHS) is recognized as an efficient strategy for reducing health inequalities^4,5^. Regarding chronic conditions, among which hypertension and diabetes stand out, the FHS promotes the diversification of access, the provision of care actions and technologies, teamwork, and the strengthening of bonds^6^.

During the pandemic, the work of Primary Health Care (PHC) teams highlighted the need to ensure continuous care to chronic health issues and prepare services for future epidemics^7^. Researchers point to the role of PHC in monitoring users, qualified listening, establishing bonds, and in the coordination of care^8–10^. Health practices provided by PHC professionals were adapted to minimize access barriers, especially among the most vulnerable groups with chronic conditions such as hypertension and diabetes^11–13^.

The pandemic presented significant difficulties for users with these conditions, such as decreased access to face-to-face consultations, absence of longitudinal monitoring of blood pressure and blood glucose, and failures in the availability of medicines and inputs essential for disease control^14,15^.

In Brazil and worldwide, the healthcare response to Covid-19 focused initially on hospital services, but much could have been done in PHC. Therefore, in this study, we sought to answer the following questions: Who were the PHC professionals that provided care to users with hypertension and diabetes during the pandemic? What practices were developed and how were they provided? What limitations and challenges were faced in the territory for the continuity of this care? Thus, the objective of this study was to analyze the work process of PHC professionals regarding health practices aimed at hypertension and diabetes during the Covid-19 pandemic.

## METHODS

This is a sub-study of the project "Expansion of strategies of testing, isolation, quarantine and telemonitoring in Primary Health Care, in communities of great social vulnerability, in coping with the Covid-19 pandemic in two Brazilian capitals" (TQT-Covid-19)^16^.

The work process of PHC professionals in a capital of Northeastern Brazil regarding health practices aimed at users with hypertension and diabetes during the Covid-19 pandemic was analyzed in this research. A Northeastern capital city was selected, structured in health regions. The largest health region was selected because it has a network of services with population coverage of 400 thousand inhabitants, thus being comparable to those of other large urban centers and municipalities of the country. The study focused on the analysis of the work process of PHC professionals, in the period from March 2020, the initial milestone of the Covid-19 pandemic, to June 2022.

A total of 35 semi-structured interviews were conducted with professionals who provided care and followed up this subpopulation in PHC, in addition to analyzing municipal documents and media productions, available with open access in official media, about Covid-19 and PHC. The documents were searched using the descriptors "Covid-19" and "Primary Health Care" on the Internet, on the official website of the Municipal Department of Health (*Secretaria Municipal de Saúde* – SMS), on the city hall's website, and on the Instagram profile of the municipality. The institutional documents of the analyzed municipality were the contingency plan for human infection of the new coronavirus (SARS-CoV-2), decrees, technical notes, and news of the city hall's website regarding the work process of PHC and professionals and/or users with hypertension and diabetes, totaling 28 documents (Chart 1).

**Chart 1:** Institutional documents related to Primary Health Care services provided in a municipality of the Northeast region, 2023.

	Document	Content related to Primary Health Care
D1	Contingency plan for human infection of the new coronavirus (SARS-CoV-2) — 03/2020.	It presents the flowcharts for the care of suspected and confirmed cases in the various levels of health care. PHC: ensure the provision of care and testing in units or referral to test collection units.
D2	Municipal Decree dated 03/16/2020	Suspension of holidays and leaves of absence of the appointed civil servants in the Municipal Department of Health; Performance of remote activities for municipal civil servants over 65 years of age.
D3	Municipal Decree dated 03/18/2020	Increase in the number of dispensed medicines and extension of the expiration date of prescriptions.
D4	Municipal Decree dated 03/18/2020	Possibility for third parties to take out medicines to avoid the displacement of older people to health units; Anticipation of the flu vaccination campaign; Establishment of remote work for municipal civil servants over 60 years of age, pregnant women, individuals with chronic and respiratory diseases, and those who use immunosuppressant drugs.
D5	Municipal Decree dated 03/19/2020	Suspension of elective dental services.
D6	Technical Note — New Coronavirus No. 01/2020, dated 03/19/2020	It establishes guidelines for organizing Primary Health Care in coping with the new coronavirus (Covid-19) in the municipality. It establishes flow to care for respiratory symptomatic patients in Primary Health Care, with user embracement, testing, notification, clinical management, referral, use of PPE, prevention measures and individual and community control, active search, and suspension of group activities. It establishes the role of CHAs, the NASF team, and the Oral Health Team in carrying out the flow for welcoming users with flu-like symptoms.
D7	Technical Note — New Coronavirus No. 02/2020, dated 03/25/2020	It updates the guidelines to organize Primary Health Care in the face of the new coronavirus (Covid-19) in the municipality.
D8	Technical Note — New Coronavirus No. 03/2020, dated 04/08/2020	It updates the guidelines on clinical monitoring every 24 h for older adult users or individuals with risk comorbidities that are suspicious cases.
D9	Technical Note — New Coronavirus — No. 05/2020, dated 04/09/2020	It establishes specific guidelines on healthcare professionals’ leave and return to activities.
D10	Decree dated 04/20/2020	On-site work leaves for employees over 60 years of age, who have a history of respiratory diseases and chronic diseases, pregnant women, or individuals who use immunosuppressant drugs.
D11	Decree dated 04/20/2020 (republished in DOM Special Edition on 04/24/2020)	Mandatory use of respirator masks in work environments for all establishments whose activities are not suspended, including municipal offices. These establishments should provide the respirator masks for their employees.
D12	DAS/APS Technical Note — New Coronavirus No. 09/2020, dated 06/23/2020	Guidelines for reorganizing the PHC work process in coping with the new coronavirus in the municipality. It establishes the flows of user embracement regarding spontaneous demand 1 and 2, with direction of professional categories for each flow.
D13	News report on the city hall's website (09/30/2020)	Health units already perform tests to detect Covid-19.
D14	News report on the city hall's website (01/30/2021)	List of Family Health Units with vaccination against Covid-19.
D15	News report on the city hall's website (02/04/2021)	List of Family Health Units with vaccination against Covid-19.
D16	News report on the city hall's website (03/20/2021)	City Hall expands health units with Covid-19 tests.
D17	News report on the city hall's website (04/10/2021)	List of Family Health Units with vaccination against Covid-19.
D18	News report on the city hall's website (04/14/2021)	List of Family Health Units with vaccination against Covid-19.
D19	News report on the city hall's website (05/03/2021)	List of Family Health Units with vaccination against Covid-19.
D20	News report on the city hall's website (10/03/2021)	List of Family Health Units with vaccination against Covid-19.
D21	News report on the city hall's website (12/09/2021)	List of Family Health Units with vaccination against Covid-19.
D22	News report on the city hall's website (01/18/2022)	List of Family Health Units with vaccination against Covid-19.
D23	News report on the city hall's website (01/21/2022)	List of Family Health Units with vaccination against Covid-19.
D24	News report on the city hall's website (02/25/2022)	List of Family Health Units with vaccination against Covid-19.
D25	News report on the city hall's website (04/30/2022)	List of Family Health Units with vaccination against Covid-19.
D26	News report on the city hall's website (05/02/2022)	List of Family Health Units with vaccination against Covid-19.
D27	News report on the city hall's website (06/09/2022)	List of Family Health Units with vaccination against Covid-19.
D28	News report on the city hall's website (06/26/2022)	List of Family Health Units with vaccination against Covid-19.

D: document; PHC: Primary Health Care; PPE: personal protective equipment; CHA: community health agent;

NASF: Family Health Support Center; DOM: Municipal Official Gazette; DAS: Health Care Board.

Source: Website of the Municipal Department of Health.

For the interviews, with a semi-structured script, a recorder was used to record the audio, which was later transcribed. All interviews were conducted in person, with a mid-level professional, a nursing technician, and a top-level professional, that is, three professionals from each of the 12 Family Health Units (FHUs) of the administrative region, at the time of data production.

The selection criterion was: professionals of the teams who worked in PHC in the 12 FHUs during the Covid-19 pandemic (March 2020 to June 2022). In one unit, there was no participation of the nursing technician.

The present study adopted the work process theory proposed by Mendes-Gonçalves^17^ as its theoretical framework. The work process theory considers as essential components the purposeful activity, that is, the work itself, the work object, and work instruments or means^17^. The object of analysis consisted of the health practices provided by PHC professionals for the comprehensive and longitudinal care of users with hypertension and diabetes, as well as the way these professionals organize their work to deliver the necessary care to this population.

The result of the work process involves the health needs (deficiencies) of users with hypertension and diabetes and the practices (object) provided by PHC professionals (agents), through which users obtain responses (products) to their health demands, whether biological, physical, mental, spiritual, or environmental. Health practices consider the objective dimensions of work and, in health services, enable a reflection on care models through the analysis of social relations, which cut across such practices, and the social representations of the agents on the health situation and its insertion in institutions^18,19^.

The health professional, in addition to a "technician of social health needs," is also a "manager of collective health work processes"^18,19^. The agents considered for this study were nurses, dentists, nursing technicians, and community health agents (CHAs). The instruments used by the agents were analyzed in the FHUs, considering equipment availability and adequacy, physical structure, and immaterial knowledge such as training, experiences, and practices of professionals. The work instruments can synthesize the qualities of the object and the project to achieve the intended effect^17^.

The interviews were anonymized, transcribed, and reviewed using the NVIVO 11 software. The empirical material produced was organized and analyzed in the same software. All the collected material was organized, coded, and analyzed according to the categories of the health work process reference^17^: agents, objects, and instruments. Triangulation of data was adopted as a methodological strategy, articulating multiple sources and collection techniques^20^. Data systematization and analysis were carried out as per the analysis plan (Chart 2).

**Chart 2:** Matrix analysis of category and conceptual elements that compose the work process of professionals of Family Health Teams in a municipality of the Northeast region, 2023.

Category of analysis	Conceptual elements	Questions and remarks on the script	Checking sources
Work process	Agents	What is your profession? What is your highest degree? Date of birth? Sex? Employment relationship with the municipality. Date of employment relationship? Skin color (self-reported)? Do you have another occupation besides working in the health unit? What is the approximate family income (in minimum wages)?	Interview with the professionals of the Family Health Units
	Object	What are the programmatic actions developed in this unit? How did the unit organize itself to perform actions not related to Covid-19 concomitantly with actions focused on Covid-19 (investigate Covid and non-Covid user embracement and programmatic actions — how did the teams organize themselves)? What happened to CHAs’ work during the pandemic? In the documents, did you observe the protocols and definitions of the municipal government regarding chronic conditions of hypertension and diabetes in the network flow?	Interview with professionals of the Family Health Units SMS documents published from March 2020 to June 2022
	Material and immaterial instruments	Was there sufficient and adequate PPE? Mention some experiences prior to the employment relationship with SMS. Had you been trained on Covid-19 rapid test and self-test? Had you already have experience with online courses? Was the material knowledge — physical structure of the units, PPE, tablets, cell phones, medical records — observed in the documents? Was the immaterial knowledge — courses, training, and qualification of professionals, as well as previous experiences — observed in the documents?	Interview with professionals of the Family Health Units SMS documents published from March 2020 to June 2022

SMS: Municipal Department of Health; CHA: community health agent; PPE: personal protective equipment.

The study was approved by the Research Ethics Committee (REC) of the World Health Organization, according to ERC WHO CERC.0128A e CERC.0128B; CAAE 53844121.4.1001.5030.

## RESULTS AND DISCUSSION

### Change in the Work Process Considering the New Epidemiological Scenario

In March 2020, the beginning of the Covid-19 pandemic, the SMS launched a contingency plan that assigned to PHC the responsibility for the reorganization of its care flows in the FHUs and territories. The actions included responding to the demands of Covid-19 and PHC routine, monitoring and provision of guidance to the population, testing or referral for test collection as well as promoting preventive measures.

The analyzed territory had more than 400 thousand inhabitants served by 12 FHUs and 46 teams. According to the National Primary Care Policy, each PHC team in urban areas should serve up to 4 thousand people and each CHA, up to 750^21^. The shortage of teams, associated with high transmissibility of the disease, contributed to the overburden of professionals and prioritization of demands related to Covid-19 (Chart 3).

**Chart 3:** Health practices carried out by Primary Health Care professionals, from March 2020 to June 2022, in a municipality of the state of the Northeast region, 2023.

Health units	Actions to respond to Covid-19				Care to chronic conditions — hypertension and diabetes			
	Testing	Surveillance actions	Vaccine	Evidence	Planning	Programmatic actions carried out	Activities	Evidence
FHU 1	Carried it out	Contact tracing was not carried out; Telemonitoring was performed; Active search was not carried out.	Was a vaccination center against Covid-19	"No, contact tracing was not carried out. We did [the test] in those who came there, you know? We made suggestions. For example, if someone came, a wife, we suggested that the husband do it [the test]. If they had symptoms, we would suggest it, right? But it was not traced, no, we didn't make phone calls, trace it, not that." (I24) "We performed telemonitoring. We had some people who were working from home, who were professionals within the risk group, and they did this telemonitoring. Even one of the doctors who worked with us, he designed a telemonitoring program." (I13) "But active search for what? For Covid? […], no." (I24) "We spent more time here, in the [unit] area, organizing forms, records, this kind of thing. We served patients here at the center." (I1)	Nonstructured; Nonsystematic; Nonparticipatory.	Provision of care for Covid-19 only	Provision of care for Covid-19 only	"[Care was] suspended right at the beginning of the pandemic. Right at the beginning, we only focused on care for Covid." (I13) "There was a shift." (I24) "There was a shift, a planning of how the flow would work, so much so that here, in the unit, user embracement was carried out, what we nowadays call Flow B, as we determined the flows as Flow A and B, that is, Flow A is for symptomatic patients and Flow B, for patients who are not symptomatic respiratory." (I13) "I love health care. To help people, to guide people. If I can do anything to help, I'll do it." (I1) "The patients, I think, are well monitored, they are cared for, you know? All in all, we help them, you know? Patients don't come here just because of a dental complaint; they seek the service because of that, but if I see something different, I already refer them, you know? So, yeah, I think it's very interesting." (I24)
FHU 2	Carried it out	Contact tracing was not carried out; Telemonitoring was not performed; Active search was not carried out.	Was a vaccination center against Covid-19	"Contact tracing was unofficially done. The identification was ok, but contact tracing was done in a very informal way with the user, you know?" (I25) "We received a tablet from… [SMS program], but the program did not move forward, it weakened, there was no encouragement, it was forgotten. And amid the tests, we couldn't carry it out." (I25) "We tried [implementing] some strategies by using WhatsApp groups and such, but we had some problems, which was the discussion of the work of the community health agent […], so in the end, we ended up focusing on what everyone wanted, for everyone to feel safe, to choose performing active search for users, because at that beginning people died and nobody wanted to be responsible for it." (I25)	Nonstructured; Nonsystematic; Nonparticipatory — meeting with the team to organize the shift.	Hypertension, diabetes and procedures	Consultations, specific actions via Whatsapp, health education app for pregnant women, waiting room, prescriptions renewal, welcoming of spontaneous demand without scheduling for scheduled demand	"Yes, they were aimed at risk groups. We kept prenatal care, children's care, and patients with chronic conditions, such as diabetics, hypertensive individuals, tuberculosis, so we did not interrupt it, no. We only interrupted those more elective clinical consultations." (I25) "There was no planning, even so ‘cause someone could be absent […] it was always according to the day or the shift, who was there for user embracement and adding one more, there always had someone. But we cannot say that there was a plan for the whole week or the month, no." (I14) "The reason why I showed up to work, [because] I already have experienced some difficulties before working, with family members, you know, to need care and not be welcomed, not be cared for, and as I had the opportunity, I really took the opportunity to try to make a little difference. ‘Cause even if I did not have the conditions to provide care, I could always welcome them, even if the doctor couldn't see them, I'd say for them to come tomorrow that we would make it work, you know? To welcome them properly, that's very important." (I2)
FHU 3	Carried it out (October 2021)	Contact tracing was carried out at the time of testing of symptomatic patients, asking them to attend the unit; Telemonitoring was not performed; Active search was rarely carried out	Was a vaccination center against Covid-19	"I asked how many people there were in the house, if they had any symptom, and if someone had symptoms, I'd say for them to come here" (I15) "Our schedule was perfectly ok. In 2021, it was the vaccine; in 2022, it was practically testing; and I think that, at the end of 2022, the Covid issue was getting better, and we resumed those activities that were specific to the PSF." (I26) "Rarely [referring to active search], ‘cause most people came out of spontaneous demand really. The vast majority came due to spontaneous demand." (I26)	Nonstructured; Nonsystematic; Nonparticipatory — shift per team/day.	Hypertension, diabetes and procedures	Consultations for clinical care	"Hiperdia, which is the Brazilian program aimed at providing care for hypertensive and diabetic patients, prenatal care, family planning, children's health, the smoking program is held here, preventive cervical cancer screening, Guthrie test, vaccination, dressings, testing for sexually transmitted infections, hepatitis B and C, AIDS and syphilis, medical clinic appointments, dental appointments, PSE, tuberculosis, and leprosy." (I26) "Of the pandemic. Since the opening of the unit, we only carry out visits or registration, but visits reduced a lot, and educational activities, which is the work done by CHAs, right? Promoting prevention, health promotion and protection, that doesn't happen. I've been waiting [for it] to this day." (I3) "’Cause I love working with Primary Health Care, I really care about it, about prevention, I like it. I've never worked in a hospital, I like Primary Health Care." (I15)
FHU 4	Carried it out	Telemonitoring was performed; The other professionals reported that there was no telemonitoring or contact tracing; Active search was not carried out; Contact tracing was not carried out.	It was not a vaccination center against Covid-19	"I think it was because of the flow, which was very massive. I think it was about it [in relation to not performing telemonitoring]." (I16)	Nonstructured; Nonsystematic; Nonparticipative — meeting with nurses and the manager to organize the shift	Hypertension, diabetes and procedures	Consultations for clinical care, prescriptions renewal, health communication on the way from home to the unit	"Everything was done here at the center, but during the pandemic, a lot was suspended. But, you know, it depends on the period we're talking about. Because after the vaccinating started and cases decreased, some services were resumed, but we used to do everything, hypertension, diabetes, prenatal care, children's care, family planning, all these actions. Now, there was a suspended time." (I27) "Boy, we went through some hard times here… So, we tried, indeed, to make a planning, not everyone would gather, because as it was a pandemic, we could not get everyone together, but some did, a nurse from each team would meet with the manager and talk, whether to make a planning, organize shifts, but it was carried out somehow, yeah. We would gather to talk things through, organize shifts, you know?" (I4) "It's a matter of profile, actually. Since college, I have identified with the public health area, since my first job, I already wanted to work in Primary Health Care, in PSF, but I have had experience in management, experience in specialized care, and I like Primary Health Care, because you are not solely focused on treating the disease, but on preventing, promoting health. And that's one thing that captivates me in Primary Health Care, you can do both, you can provide care, but also promote health." (I27)
FHU 5	It was not carried out	Active search was carried out; Contact tracing was carried out at the time of testing of symptomatic patients, asking them to attend the unit; Telemonitoring was performed.	Was a vaccination center against Covid-19	"The active search was sometimes carried out, with some professionals and health agents. There was…, they had a printed form of a while ago, for positive cases. They had a printed form for follow-up […]." (I28) "I think only in a few specific cases [referring to active search]." (I5) "The professional who provided care usually identified, you know, the patient who needed monitoring. And then, professionals themselves performed the monitoring via telephone contact or the health agent, or the health agent went to the patient's home to perform this monitoring." (I28)	Nonstructured; Nonsystematic; Nonparticipatory — team meetings to organize shifts.	Hypertension and diabetes	Reduced consultations and waiting room concerning Covid-19 topics	"We reduced the number of consultations. And then there was the shift issue, right? It also depended on the day, you know? Depending on the shift, there were some professionals focused on the care of symptomatic respiratory patients and others were doing the same routine care." (I28) "It's because, actually, there's not much hypertension and diabetes like before, when we had Hiperdia. In fact, it's an appointment with the same clinician, and they also see hypertensive and diabetic patients." (I28) "We always discussed it in team meetings." (I5) "It's the issue with wages [laughs] and also because, like, the way […] to work like that, be closer to the community. The actions that are inserted in the health unit, I think it's very interesting, this direct contact we have with the patient." (I17)
FHU 6	Carried it out (for a period of one month)	Active search was carried out in specific cases; Contact tracing was not carried out; Telemonitoring varied.	Was a vaccination center against Covid-19	"You mentioned… [the SMS program] and I remember that, indeed, there was one…, but it started at the end. Not in the end, but at a time when the pandemic was not so severe. It took a long time for it to start, but we had the…, sometimes, we had patient monitoring. It was not a constant thing." (I29) "Except when the reference contacted the unit and asked to do an active search for this patient. But it was nothing like that…, for example, if we knew that a certain patient had Covid; it was nothing certain, you know." (I18)	Nonstructured; Nonsystematic; Little participatory — team meeting once. The shift was organized by the manager.	Hypertension and diabetes	Consultation — spontaneous demand	"In the beginning, to establish how this user embracement would take place, there was a meeting; but afterwards, a pre-organized shift was followed." (I18) "Of routine programs, I think almost none. Probably, planning and obstetrics, for prenatal follow-up. I believe that only these… [referring to programs for children's care, diabetes, tuberculosis, oral health] yes, it was interrupted." (I29) "We had guidance, what guidance to provide for users upon their arrival, for instance, Hiperdia patients, when they arrived, if they needed a prescription, if their prescription had expired, they were seen in user embracement, so the doctor could be informed and renew this prescription, not always requiring a consultation. I think it interfered a lot in the care for these patients." (I18) "Oh, I'm passionate about health care." (I6)
FHU 7	Tests were not performed	Active search was carried out; Telemonitoring was performed; Contact tracing was carried out at the time of telemonitoring.	Was a vaccination center against Covid-19	"Right, as it was determined that CHAs would only have to visit patients in cases of actual priority. They did not have to make periodic visits. Then, it was very restricted indeed, [the visits would be made] only if they knew about a suspected case there." (I30) "There was for the issue of symptomatic respiratory patients. Even before TQT, testing, we did it. Every 48 hours, we called patients. At first, it was 14 days of isolation. In this period of 14 days, every 48 hours, we called patients to know how they were doing, for instance, if someone in their home presented with any symptom, got worse, how was the patient during the 14 days of isolation." (I19)_ "The same team. Usually, the traced contacts were, like, for example, we called a patient and the patient said that the daughter presented with symptoms, then we traced this daughter, right, and explained [the situation], you know, so they could come to the unit to be examined." (I19)	Nonstructured; Systematic; Participatory — meeting with all professionals to organize the shift and depending on the need.	Provision of care for Covid-19 only	Provision of care for Covid-19 only	"Pretty much nothing, to be honest. We were completely focused on Covid We suspended medical care, even following the guidance of the Department of Health. At that time of the pandemic, we weren't supposed to see patients." (I19) "There was a meeting once a week [about] what wasn't working, we would fix it, especially the doctors and nurses." (I7) "In fact, in practice, no, because, like I said, we were all focused on Covid, so it turned out that the other programs were really left aside." (I19) "When I first started, I was smitten. At first, I found it boring, but I started to see the issues focused on prevention, education, and then I fell in love [with it]." (I19) "Because that was the area I identified with the most." (I30)
FHU 8	It was not carried out	Active search was carried out on demand; Telemonitoring was performed; Contact tracing was carried out at the time of telemonitoring.	Was a vaccination center against Covid-19	"There was a monitoring system, the staff did it, nurses, dentists. I don't know if the doctor was part of this monitoring, of calling people to know how they were doing, the symptoms, if there was someone else in the house who had presented with symptoms, if they had taken the test and tested positive." (I20) "Yes, both them and myself, I carried out many visits by cell phone, [by] calling the patient. I called them to know how they were, I was monitoring them." (I8) "In some cases, yes, we asked the community agent to carry out this active search, right? It was more difficult when patients weren't…, when their area had no agent. That's quite harder, you know? Then we tried to call, we really did…, but visiting them was difficult sometimes." (I31)	Nonstructured; Nonsystematic; Little participative — meetings.	Urgent demands	Consultations, procedures and pharmacy	"Prenatal care was maintained, care for more urgent things… but, for example, prescriptions for hypertension and hypoglycemia… for, like, hypertensive and hypoglycemic patients, they were extended without having an expiration date, right, which is usually 6 months… […] basically, we were seeing symptomatic patients, prenatal care, people with a more urgent complaints. Cervical cancer screening exams were also suspended. Everything that was no longer urgent had to be suspended. Educational activities were also suspended." (I31) "There were some moments. But, like, in fact, much of what we did was… it has already been determined by the district itself. We had meetings, but very occasional meetings, as we barely had time to have these meetings with everyone. […] But I remember that a lot was determined by the district: how is it going to work, who are we going to see, how many patients approximately." (I31) "And, you know, that compelled me, because taking care of people is what I love, right? […] you can change people's lives in relation to their own health […] small things, you know? Guidance regarding the care for oneself, which some people don't have, sometimes, but when you get there, you start taking care of that family, that person, those people, it changes everything. It is fulfilling." (I8)
FHU 9	Carried it out	Telemonitoring was not performed — lack of HR; Active search was carried out; Contact tracing was carried out at the time of testing symptomatic patients, requesting them to attend the unit.	Was a vaccination center against Covid-19	"Because here, in the District, there was a policy…, there still is, right: where you get a vaccine for Covid, you don't test for it. So we interspersed this reference: sometimes it was a vaccine reference, sometimes it was a test reference." (I32) "We had it, like, at the time of the appointment, we identified the contacts and called them, right." (I32) "And then… But, you know, they did not always come. But we called them…, we even gave them a term, which the patient signed, with the name of all contacts, saying that they had a commitment to attend the unit." (I32)	Nonstructured; Nonsystematic; Participatory — meetings.	Diabetes and hypertension.	Consultations, dressings, pharmacy, exams to schedule in the system.	"So, the scheduling was online, for consultations that continued to be in person, because there were hypertensive patients for whom prescription renewal was not enough, diabetics who require a closer care. Now, there were others who had recently been seen, with recent exams, and we could perform teleconsultation. Or, then, in this first stage, we would ask for an exam via teleconsultation, right, we would leave all the exams and prescriptions separated and, once the patient had the results, then we would schedule a face-to-face appointment, which was a more complete consultation." (I32) "So, I carried out the initial plan, the manager asked me to do so. I made an initial plan and then gathered the team. There were representatives of NASF, CHAs, dentists, doctors. And then, we discussed what would be feasible and what should be changed. In the end, we did…, we passed it on to the rest of the unit, per category, team, and adapted it, also according to the demand. Everyone could have a say." (I32) "Me? Unemployment. I was unemployed, I got this job and here I am." (I9)
FHU 10	Carried it out	Telemonitoring was performed; Contact tracing was not carried out; Active search was not carried out.	Was a vaccination center against Covid-19	"That time, I can't explain, no. But I know that positive patients were immediately telemonitored, all this kind of consultation. Now, for how long […]." (I21) "It was not carried out, no, ‘cause they said that the patients didn't let them enter their houses. There was also that issue of PPE, that all of them had to be protected accordingly, there was the issue of patients’ refusal." (I21). "[Regarding active search] So, we do… The actual figure was 750, so we always prioritized, you know, those cases that… As we already have a certain contact of years with the community, so we already know who the most vulnerable, most exposed people are, so these were the people that we always […]." (I10) "[Regarding contact tracing] No, I think it was only the patients who were positive." (I21)	Nonstructured; Nonsystematic; Nonparticipatory — shift per professional category.	Does not mention hypertension and diabetes	Consultations	"Here in the unit, basically, I was the only nurse… so, I ended up on testing…, and there was a shift between dentists and me. So, we saw all kinds of cases, but it was through shifts. One day we were working on Covid-related care, the other we were focused on our own appointments." (I33) "I think that… vaccination worked. There were days that it worked and other days that it didn't. We did not have much contact with this part of leprosy and tuberculosis, because that was the responsibility of the doctor. We did not have much contact [with it], to know whether was it working or not […], they had some [referring to the doctors] who stayed there in the room, seeing pregnant women. This part of tuberculosis and leprosy we do not know. (I21) "The day of the week was the day of the team, that's how it worked. […], for example, Tuesday was my team's day, so we had that responsibility on Tuesday. On Wednesday, it was another team." (I10) "Look, I think one of the main reasons is the issue of demand… so, the Primary Health Care demand is much bigger, because you have a larger amount of PSF, of Health Centers. So, I came to Primary Health Care mostly because of this and for identifying with it. I rather work with programmed actions, so I can have control of them, than, for example, with BP. With BP, nothing is controlled, every day is a different dynamic, you know, in the hospital. So, that's why I prefer Primary Care." (I33)
FHU 11	Carried it out	Telemonitoring was performed; Active search was not carried out — lack of CHAs; Contact tracing was not carried out.	Was a vaccination center against Covid-19	"From four to five days, we did the active search to know if the patient…, how the patient was." (I11) "Yes. After the appointment, all suspected or confirmed patients were sent to me, then I did this monitoring, telemonitoring, within 14 days. And then, as soon as they were tested and received the result, I ended it, if they already had no symptoms. If symptoms persisted, I continued performing it." (I22) "We did it for a while, but it wasn't continuous. Vaccine and testing were always carried out, the Department of Health made rotations. So, for some time, we had the vaccine; another time, we had testing. And the Department created strategies and made rotations among the units." (I34)	Nonstructured; Nonsystematic; Nonparticipatory — shifts organized by the manager.	Chronic patients	Consultations and prescriptions renewal	"So, you mean the provision of care, am I right? We had a phase of the pandemic [in which] the Department restricted the number of consultations […], as this unit was inaugurated in 2020, it was inaugurated in the pandemic boom. […] We were told to focus only on Covid…, so we examined these symptomatic patients. We saw patients of other programs, but only chronic patients, you know, who had [chronic conditions], needed a prescription, who received some continuous treatment. But the prevention part, at that moment, we stopped doing it so the team could be focused on the pandemic. After controlling this situation and with the beginning of vaccination, cases were getting milder, we began to have more availability to resume prevention actions of Primary Health Care." (I34) "Everyone had to work, you know? The manager organized the shifts for providing care, who was going to be on the shift for welcoming respiratory symptomatic patients and who would be in the testing. And all the professionals were involved in this shift." (I34) "Damn, you got me! How have I chosen it? In fact, I didn't choose it. […] when I applied for the public tender, in the selection process, I chose direct management, but with 40 hours, aiming at working in urgency and emergency. But then, when I got in, they sent me to Primary Health Care. I didn't choose it." (I22)
FHU 12	Tests were not performed	Active search was not carried out; Contact tracing was not carried out; There was an attempt to perform telemonitoring.	Was a vaccination center against Covid-19	"We tried [in relation to telemonitoring], right, but there was only one phone for the whole unit. It didn't work." (I35) "No, we didn't have it [regarding contact tracing]. Guidance was provided by the doctor, in case patients had contact with someone, he asked them to take the test somewhere." (I23) [In relation to active search] It's like I said, right, we tried, but we couldn't have proper access to patients, ‘cause we only had one cell phone. And then this phone stopped working. That was it, we couldn't […]." (I35)	Nonstructured; Nonsystematic; Non-participatory — shift.	Hiperdia	Consultations	"[…] And then in the most urgent care, the demand here was […] the patients were decompensated, so, you know […] many decompensated patients, with hypertension and diabetes, mental health, this kind of thing, so they needed a closer follow-up, because they did not have a health unit here. So, we focused on them. And then, some cases of the pandemic increased, and we already provided care for those too, the Covid cases. It was like that, the usual demands, right, and the Covid demands." (I35) "There was a shift. Each day there was a responsible team, each day a doctor, and once a week there was rotation." (I35) "Girl, when I applied for the public tender, I didn't even believe I was going to work. At the moment, even when I did it, I was unemployed, I needed a job, and when the news came, my God, I couldn't believe it. And today, here I am, right? I like what I do, I like to be an agent, that's it." (I12)

FHU: family health unit; I: interviewee; SMS: Municipal Department of Health; CHA: community health agent; PSF: Family Health Program; PSE: School Health Program; TQT: Project "Expansion of strategies of testing, isolation, quarantine and telemonitoring in Primary Health Care, in communities of great social vulnerability, in coping with the Covid-19 pandemic in two Brazilian capitals"; NASF: Family Health Support Center; BP: blood pressure.

In the FHUs, users were initially received by the spontaneous demand team and, based on the initial assessment, they were directed to two care flows: symptomatic respiratory patients (flow 1) or other demands, including hypertension and diabetes (flow 2). Symptomatic respiratory patients had priority for medical care, as well as people who had contact with suspected or confirmed cases, according to municipal documents (D1 and D6). The division of flows aimed to reduce the risk of contagion of users and professionals^22^.

Extended prescription validity for continuous-use medications, expanded medication dispensing (D3), and remote work for civil servants with comorbidities were some of the measures adopted to promote social distancing. Faced with a scenario for which PHC was not prepared, the management sought to normalize new practices through official documents. A PHC system structured according to the needs of the population and aligned with its attributes was essential for an effective response to Covid-19. The new context, with the start of the pandemic, caused a sudden change in the routine of PHC professionals, as it was necessary to adapt quickly to continue providing care to users of their territory.

### Who Were the Agents of the Work Process?

Professionals of the PHC units acted as agents of the work process in the development of health practices aimed at users with hypertension and diabetes (Chart 4).

**Chart 4:** Profile of the agents who worked in the Family Health Program, in a municipality of the state of Bahia, 2023.

Agents	Position (role)	Highest degree	Type of employment relationship with the municipality	Year since working in the current position	Age (years)	Sex	X…	Self-reported skin color	Family income in MW	Another occupation
I1	CHA	High school	NI	NI	40 to 49	Woman		White	1 to 2	Yes
I2	CHA	High school	NI	NI	50 to 59	Man		Black	1 to 2	No
I3	CHA	Higher Education	NI	NI	40 to 49	Woman		Black	1 to 2	No
I4	CHA	Technical school	Statutory	1998	50 to 59	Woman		Black	1 to 2	No
I5	CHA	Higher Education	Statutory	2003	50 to 59	Woman		Black	3	No
I6	CHA	Higher Education	NI	2008	50 to 59	Woman		Mixed-race	1 to 2	No
I7	CHA	High School and Technical CHA	Statutory	2007	50 to 59	Woman		Mixed-race	4 to 5	Yes
I8	CHA	High school	Statutory	2007	50 to 59	Woman		Mixed-race	1 to 2	No
I9	CHA	High school	Statutory	2007	> 60	Woman		Black	1 to 2	No
I10	CHA	High school	Statutory	2007	50 to 59	Woman		Black	1 to 2	No
I11	CHA	High school	Statutory	2011	50 to 59	Woman		Black	1 to 2	No
AI2	CHA	High school	Statutory	2007	50 to 59	Woman		Black	1 to 2	No
I13	Nursing technician	Higher Education, Specialization degree	NI	NI	30 to 39	Woman		Mixed-race or Black	4 to 5	Yes
I14	Nursing technician	Higher Education, Specialization degree	NI	NI	40 to 49	Woman		Mixed-race	3	No
I15	Nursing technician	Higher Education, Specialization degree	NI	NI	50 to 59	Woman		Mixed-race	3	No
I16	Nursing technician	Higher Education, Specialization degree	NI	NI	30 to 39	Woman		Black	4 to 5	Yes
I17	Nursing technician	Higher Education	REDA	2018	40 to 49	Woman		Mixed-race	3	No
I18	Nursing technician	Technical school	Statutory	2013	40 to 49	Woman		Black	3	No
I19	Nursing technician	Higher Education	Statutory	2013	40 to 49	Woman		Mixed-race	4 to 5	Yes
I20	Nursing technician	Higher Education, Specialization degree	Statutory	2013	40 to 49	Woman		Mixed-race	3	No
I21	Nursing technician	Higher Education	Statutory	2014	40 to 49	Woman		Mixed-race	4 to 5	No
I22	Nursing technician	Technical school	Statutory	2018	40 to 49	Woman		Mixed-race	4 to 5	Yes
I23	Nursing technician	Technical school	REDA	2019	40 to 49	Woman		Black	3	No
I24	Dentist	Higher Education, Specialization degree	NI	2020	50 to 59	Woman		White	> 10	Yes
I25	Nurse	Higher Education, Specialization degree	Statutory	2003	40 to 49	Man		Mixed-race	6 to 10	Yes
I26	Nurse	Higher Education, Specialization degree	NI	NI	40 to 49	Woman		Mixed-race	> 10	Yes
I27	Dentist	Higher Education, PhD	NI	NI	40 to 49	Woman		Mixed-race	NI	Yes
I28	Nurse	Higher Education, Specialization degree	REDA	NI	30 to 39	Woman		White	4 to 5	No
I29	Dentist	Higher Education, Specialization degree	Statutory	2014	30 to 39	Man		Mixed-race	4 to 5	Yes
I30	Nurse	Higher Education, Specialization degree	Statutory	2013	40 to 49	Woman		Mixed-race	6 to 10	No
I31	Nurse	Higher Education, Specialization degree	Statutory	2014	30 to 39	Woman		Mixed-race	6 to 10	Yes
I32	Nurse	Higher Education, Specialization degree	Statutory	2013	30 to 39	Woman		White	6 to 10	No
I33	Nurse	Higher Education, Specialization degree	Statutory	2012	30 to 39	Woman		White	6 to 10	No
I34	Nurse	Higher Education, Master's degree	Statutory	2015	30 to 39	Woman		Black	NI	No
I35	Nurse	Higher Education	Statutory	2013	30 to 39	Woman		White	4 to 5	No

I: interviewee; CHA: community health agent; MW: minimum wage; NI: no information; REDA: temporary employment contract with public service.

We analyzed interviews with 35 PHC professionals (CHAs, dentists, nurses, and nursing technicians). Most of them reported to be mixed-race or Black (82.8%) and 17.1%, white. Women predominated (91.4%) among interviewees. Regarding employment relationship, 60% of the professionals had a statutory employment relationship, 8.5% had a temporary employment contract, and 31.5% did not inform it. Regarding time working in the same unit, 65.7% of the professionals reported to be in the same unit for over ten years, 5.7% up to five years, and 28.6% did not inform it.

The predominant age group was 40 to 49 years (40%), followed by 50 to 59 years (31.4%), and 30 to 39 years (25.7%). Only one professional was over 60 years of age. According to the municipal decree (D2), civil servants over 65 years of age, with chronic conditions, pregnant, or who made use of immunosuppressant drugs were assigned for remote work, especially via telemonitoring. Among the 35 professionals, only one was relocated to this function and another was relocated to testing outside the unit.

### Duty and Covid-19: Practices Aimed at Users with Hypertension and Diabetes in PHC

With the reorganization of the work process (D12), professionals, working within the perspective of health surveillance, should remotely monitor users with hypertension and diabetes served by the teams, registering it in medical records. Based on this monitoring, professionals should develop actions to monitor health conditions, organize prescription renewals, and provide guidance for stable users, through teleconsultations, face-to-face consultations, or home visits (Chart 3).

Considering the increase in Covid-19 incidence, the reorganization of care provision in FHUs, the illness of professionals, and the insecurity for some activities, the flow of needs imposed by the disease were prioritized. Thus, in the interviews, we evidenced the suspension or reduction of the number of clinical care and the longitudinal follow-up of users with hypertension and diabetes (Chart 3).

What distinguishes PHC professionals apart is the bond established with users and in their qualified insertion in the registered territory. In this context, CHAs play a strategic role, especially in the care for people with hypertension and diabetes. Their performance allows continuous approximation of users, enabling care practices that go beyond the use of medicines, such as health education initiatives aimed at strengthening autonomy and self-care, fundamental in the face of restrictions imposed by the pandemic.

However, this potential of PHC was compromised by structural changes, either in the redirection of CHAs to the internal work of the units, or in the prioritization of deficiencies related to the pandemic. We observed that, in this territory, the increase in the number of FHUs and teams was not proportional to the number of CHAs. On the contrary, there was a reduction of these professionals in the teams and redirection of their functions to other units. This unbalance weakened the main differentiating factor of PHC, limiting the capillarity of continuous care actions, which are essential to control hypertension and diabetes.

In addition to this continuous and longitudinal follow-up, there were health practices that, at that time of the pandemic, were not emphasized by professionals, such as integrative and complementary actions, support to self-care, and monitoring by virtual means, or even visits of CHAs (Chart 3).

The lack of follow-up of the clinical conditions of users with hypertension and diabetes may have aggravated their health over time. Recomposing PHC teams is an essential current challenge for the longitudinal follow-up of users with hypertension and diabetes, in addition to the strategy of risk stratification of these users and the planning of actions, which favor the continuity of care during and after the pandemic^7^.

It was evidenced in the interviews that care practices for users with hypertension and diabetes were based on spontaneous demand for the units (Chart 3). The organized provision of health practices, based on risk stratification and user monitoring, was not pointed out in professionals’ statements, nor were there reports of practices for promoting health and preventing diseases related to hypertension and diabetes (Chart 3).

Furthermore, we found no consistent pattern in the provision of health practices for users with hypertension and diabetes regarding the time of employment relationship and performance in the same function by the social agents participating in the study (Chart 5).

**Chart 5:** Ratio of agents, instruments, and products of the work process of professionals of the family health teams of a municipality of the state of Bahia, 2023.

FHU	Position (role)	Agents	Highest degree	Type of employment relationship with the municipality	Time in the current position	Another occupation	Implementation of the first team CNES	FHT:OHT CNES ratio	Practices aimed at DM/SAH
1	CHA	I1	High school	NI	NI	Yes	2018	4:4	No, Covid-19 only
	Nursing technician	I13	Higher Education, Specialization degree	NI	NI	Yes			
	Dentist	I24	Higher Education, Specialization degree	NI	2020	Yes			
2	CHA	I2	High School	NI	NI	No	2013	4:4	Yes, spontaneous demand
	Nursing technician	I14	Higher Education, Specialization degree	NI	NI	No			
	Nurse	I25	Higher Education, Specialization degree	Statutory	2003	Yes			
3	CHA	I3	Higher Education	NI	NI	No	2020	4:4	Yes, but reduced
	Nursing technician	I15	Higher Education, Specialization degree	NI	NI	No			
	Nurse	I26	Higher Education, Specialization degree	NI	NI	Yes			
4	CHA	I4	Technical school	Statutory	1998	No	2011	4:4	Suspended at first
	Nursing technician	I16	Higher Education, Specialization degree	NI	NI	Yes			
	Dentist	I27	Higher Education, PhD	NI	NI	Yes			
5	CHA	I5	Higher Education	Statutory	2003	No	2015	2:2	Yes, but reduced
	Nursing technician	I17	Higher Education	REDA	2018	No			
	Nurse	I28	Higher Education, Specialization degree	REDA	NI	No			
6	CHA	I6	Higher Education	NI	2008	No	2005	4:1	Suspended at first
	Nursing technician	I18	Technical school	Statutory	2013	No			
	Dentist	I29	Higher Education, Specialization degree	Statutory	2014	Yes			
7	CHA	I7	High School and Technical CHA	Statutory	2007	Yes	2013	4:4	No, Covid-19 only
	Nursing technician	I19	Higher Education	Statutory	2013	Yes			
	Nurse	I30	Higher Education, Specialization degree	Statutory	2013	No			
8	CHA	I8	High school	Statutory	2007	No	2006	3:3	Yes, by appointment, urgent demands only
	Nursing technician	I20	Higher Education, Specialization degree	Statutory	2013	No			
	Nurse	I31	Higher Education, Specialization degree	Statutory	2014	Yes			
9	CHA	I9	High school	Statutory	2007	No	2004	4:4	Yes
	Nurse	I32	Higher Education, Specialization degree	Statutory	2013	No			
10	CHA	I10	High school	Statutory	2007	No	2014	5:5	No, Covid-19 only
	Nursing technician	I21	Higher Education	Statutory	2014	No			
	Nurse	I33	Higher Education, Specialization degree	Statutory	2012	No			
11	CHA	I11	High school	Statutory	2011	No	2020	4:4	Yes
	Nursing technician	I22	Technical school	Statutory	2018	Yes			
	Nurse	I34	Higher Education, Master's degree	Statutory	2015	No			
12	CHA	I12	High school	Statutory	2007	No	2020	4:4	Yes
	Nursing technician	I23	Technical school	REDA	2019	No			
	Nurse	I35	Higher Education	Statutory	2013	No			

FHU: Family Health Unit; I: Interviewee; CHA: Community Health Agent; NI: No information; REDA: Temporary employment contract with public service; FHT: Family health team; OHT: Oral Health Team; CNES: National Registry of Health Establishments; DM: Diabetes mellitus; SAH: Systemic Arterial Hypertension.

The work process of PHC professionals in the care provided to users with hypertension and diabetes encompasses a set of practices that extend beyond clinical consultations by doctors and nurses. This involves an expanded and continuous follow-up, which includes actions aimed at promoting therapeutic adherence, stimulating regular physical activity, encouraging healthy eating and stress management, as well as facing psychological difficulties, intensified by the pandemic restrictions. These practices contribute to strengthening self-care and self-knowledge, favoring the construction of a better quality of life and autonomy of users in managing their chronic condition.

### Limits and Challenges of the New Scenario in PHC: Discrepancies between What is Intended and What is Achieved

In Brazil, there was an increase in the incidence of users with hypertension and diabetes during the pandemic, aggravating the scenario of chronic conditions, and constituting the so-called fourth wave of the pandemic, which may bring exacerbations for many years to follow^23^. Thus, the object of the work process of PHC professionals was more directed to address the demands of Covid-19, putting the health needs that coexisted in the territory in the background.

Regarding the planning for actions in the health units, overall, we verified a non-programmatic moment, with actions aimed at organizing shifts, in which the team was divided between "Covid actions" and "non-Covid actions," and also in a non-systematic way, because they were carried out according to the routine of the unit and the demands that emerged, with little or no participation of the professionals by the team in discussing the best strategies or working conditions.

In a health crisis, actions must be aimed at identifying high-risk patients and developing a plan that organizes and directs health practices for users with chronic conditions^7^. Planning, in health management, is a way to design, organize, and monitor proposals in order to operationalize institutional decisions^24^. Thus, we verified a structured and systematic planning in the actions at the level of the SMS, but this was not evidenced in the statements of professionals regarding planning and practice in FHUs (Chart 3).

The "stay-at-home" requirements of the pandemic led users of FHUs to stay away from the services and to the discontinuity of face-to-face longitudinal care for those with chronic conditions, but new technologies were inserted in the pandemic scenario, enabling new forms of access^11,16^. To address the rapid change in the work process in PHC, instruments to support practices and prepare professionals and, especially, funding to support these changes, were necessary^9^.

From this perspective, when analyzing the instruments that would be used by professionals as means of transformation for the final product, we found inconsistences regardinsg the training of agents, as some of them reported training on Covid-19 and others reported that the knowledge was acquired by their own means, through information shared in the media and passed on by the units’ management. Another gap was the availability of personal protective equipment such as N95 respirators (Chart 3).

Institutional documents referred to the new technological tools as strategies to minimize the risks to professionals and avoid leaving users without care, especially those from strategic groups, which included users with chronic health conditions. However, the SMS attempt to expand access to users in PHC was not equitably carried out in the 12 units of the administrative region. The care practices of users with hypertension and diabetes in PHC require changes from both professionals and managers prepared to act in providing care and open to break with the hospital-centric model and public policies that subsidize a new care model, in addition to users who are empowered and co-responsible for their health process^6^.

In a scenario in which PHC had uncovered areas, CHAs focused on bureaucratic work within the units, a reduction of face-to-face or virtual consultations, prescriptions with extended expiration date without checking the control of parameters, and lack or insufficiency of equipment to monitor this subpopulation, in addition to professionals’ own fear of becoming contaminated, the limitations in the work process were vehemently evidenced in the interviews of PHC professionals (Chart 3).

Health practices aimed at users with hypertension and diabetes were based on spontaneous demand, especially for prescription renewal. Practices of disease prevention and health promotion were not identified in the evidence of this study. The agents of this process must have conditions to know their users and the territory in which they are inserted. To this end, it is necessary to expand the health teams with CHAs and the number of professionals, in addition to structuring the work process. Social agents are moved by a will to change the final product of their work process.

Impairments to monitoring and access to PHC for users with hypertension and diabetes in the pandemic was also reported in other locations, which imposed on workers the readjustment of their work process^25,26^. Health practices based on spontaneous demand were evidenced, with weaknesses in monitoring users served by teams and focus on prescriptions renewal, similar to experiences in other Brazilian territories^4,25,26^.

The Covid-19 pandemic showed a change in the final product of the work process, which was more directed to the effects of the pandemic than related, comprehensively, to the improvements in the health of the population contemplated by the PHC perspective.

## CONCLUSIONS

Given the new reality imposed by the Covid-19 pandemic, it is evident that this period caused changes in the health practices developed by PHC professionals. At that moment, it was necessary for these agents to reinvent themselves, even with little skill/resource for the new reality and with high demand for the new health needs imposed by the that scenario^4,25^.

Users with hypertension and diabetes stayed away from the units, either because of fear of contamination or because the redirection of professionals’ attention to the Covid-19 demands; nonetheless, with the progression of the pandemic, the rollout of the vaccine and, consequently, the decrease in the incidence of cases, care was slowly resumed, but with limited features.

It is noteworthy that health practices aimed at users with chronic conditions, such as hypertension and diabetes, remain fragmented and should be reorganized/qualified, but based on equity, with systematic planning, monitoring, and in-depth knowledge of their territory, in such a way that comprehensive and problem-solving access can be offered to this population, which remains "isolated," despite the end of the pandemic.

## Data Availability

The research data not included in the article are unavailable.
