# Supplementary material for: Covid-19 and chronic conditions: lessons to strengthen care in primary health care
Source: Rev Saude Publica. 2026 May 1;60(Suppl 1):e5s. doi: 10.11606/s1518-8787.2026060006917 (PMC13143267; doi:10.11606/s1518-8787.2026060006917)
Supplement: Translation

# Translation: Covid-19 e condições crônicas: lições para fortalecer o cuidado na atenção primária

**Authors:** Marina Souza Vieira, Thais Regis Aranha Rossi, Carla Maria Lima Santos, Laio Magno, Ines Dourado

**Affiliations:** I Universidade do Estado da Bahia. Departamento de Ciências da Vida. Salvador, BA, Brasil; II Secretaria de Saúde do Estado da Bahia. Salvador, BA, Brasil; III Universidade Federal da Bahia. Instituto de Saúde Coletiva. Salvador, BA, Brasil; IV Universidade Estadual de Feira de Santana. Departamento de Saúde. Feira de Santana, BA, Brasil; V Fundação Oswaldo Cruz. Instituto Gonçalo Moniz. Salvador, BA, Brasil

**Keywords:** Modelo de Cuidado de Condições Crônicas, Covid-19, Preparação para Pandemia, Atenção Primária à Saúde

## Abstract

**OBJETIVO:**

Analisar o processo de trabalho de profissionais da Atenção Primária à Saúde (APS) quanto às práticas em usuários com hipertensão e diabetes durante a pandemia de Covid-19 em uma capital do Nordeste brasileiro.

**MÉTODOS:**

Foram realizadas 35 entrevistas semiestruturadas com profissionais que atenderam e acompanharam essa subpopulação na atenção primária à saúde, assim como foram analisados documentos municipais e reportagens da Secretaria Municipal de Saúde acerca da Covid-19 e da APS. Os critérios para participar do estudo estiveram relacionados a trabalhadores das equipes de APS que atuaram na pandemia de Covid-19 na mesma unidade de Saúde da Família. Os dados foram analisados segundo referencial do processo de trabalho em saúde de Mendes-Gonçalves.

**RESULTADOS:**

A pandemia impôs mudanças significativas nas práticas de cuidado da APS. Observou-se priorização da demanda espontânea, fragilidades no monitoramento contínuo dos usuários, ênfase na renovação de receitas e atuação em territórios extensos, com áreas descobertas, pela Estratégia Saúde da Família.

**CONCLUSÃO:**

As práticas de saúde aplicadas aos usuários com condições crônicas, como hipertensão e diabetes, seguem fragmentadas e precisam ser retomadas, porém pautadas na equidade, com planejamento, monitoramento e conhecimento do seu território para que se possa ofertar o acesso integral e resolutivo à população que continua "isolada", mesmo com o fim da pandemia.

## INTRODUÇÃO

No Brasil, o Sistema Único de Saúde teve papel importante no manejo e no controle da Covid-19^1^ contudo, o período foi marcado por manifestações de negacionismo quanto à gravidade da crise sanitária, além de divergências técnicas na condução do governo federal, o que gerou polarizações políticas e sociais em meio à maior emergência de saúde pública do século XXI^2,3^.

A Estratégia Saúde da Família (ESF) é reconhecida como uma estratégia eficiente na redução das iniquidades em saúde^4,5^. Em relação às condições crônicas, entre as quais se destacam a hipertensão arterial e a diabetes, a ESF promove a diversificação do acesso, a oferta de ações e tecnologias de cuidado, o trabalho em equipe e o fortalecimento de vínculos^6^.

Durante a pandemia, o trabalho das equipes da Atenção Primária à Saúde (APS) evidenciou a necessidade de garantir atendimento contínuo aos problemas crônicos de saúde e preparar os serviços para futuras epidemias^7^. Publicações apontam o papel da APS no acompanhamento de usuários, na escuta qualificada, no vínculo e na coordenação do cuidado^8–10^. As práticas de saúde ofertadas pelos profissionais da APS foram adaptadas para minimizar as barreiras de acesso, especialmente entre os grupos mais vulneráveis e com condições crônicas, como hipertensão e diabetes^11–13^.

A pandemia trouxe dificuldades significativas para os usuários com essas condições, como diminuição do acesso a consultas presenciais, ausência de acompanhamento longitudinal dos níveis pressóricos e glicêmicos e falhas na disponibilização de medicamentos e insumos essenciais para controle das doenças^14,15^.

No Brasil e no mundo, a resposta sanitária à Covid-19 foi centrada nos serviços hospitalares, mas no âmbito da APS, muito poderia ter sido feito. Diante disso, este estudo buscou responder a: quem foram os profissionais da APS que ofertaram as práticas de saúde aos usuários com hipertensão e diabetes durante a pandemia? Quais práticas foram desenvolvidas e como foram ofertadas? Quais limitações e desafios foram enfrentados no território para a continuidade desse cuidado? Assim, o objetivo deste estudo foi analisar o processo de trabalho dos profissionais da APS no que tange às práticas de saúde voltadas às condições crônicas de hipertensão e diabetes durante a pandemia de Covid-19.

## MÉTODOS

Trata de um subestudo do projeto "Expansão das estratégias de testagem, isolamento, quarentena e telemonitoramento na atenção primária à saúde, em comunidades de alta vulnerabilidade social, no enfrentamento da pandemia de Covid-19 em duas capitais brasileiras" (TQT-Covid)^16^.

Esta pesquisa analisou o processo de trabalho dos profissionais da APS em uma capital da Região Nordeste do Brasil, no que se refere às práticas de saúde aos usuários com hipertensão e diabetes, durante a pandemia de Covid-19. Para esta pesquisa, foi selecionada uma capital do Nordeste, estruturada em regiões de saúde. Foi selecionada a maior região de saúde, por apresentar uma rede de serviços com abrangência populacional de 400 mil habitantes, sendo assim comparáveis às de outros grandes centros urbanos e municípios do país. O estudo teve como foco a análise do processo de trabalho dos profissionais atuantes na APS, no período de março de 2020, marco inicial da pandemia de Covid-19, a junho de 2022.

Foram realizadas 35 entrevistas semiestruturadas com profissionais que atenderam e acompanharam essa subpopulação na APS, além de analisar documentos municipais e produções de mídia, disponíveis em livre acesso em meios oficiais, acerca da Covid-19 e da APS. A busca pelos documentos foi feita com os descritores Covid-19 e "atenção primária" na internet, no *site* oficial da Secretaria Municipal de Saúde (SMS), no *site* da prefeitura e na rede social do município na página do Instagram. Os documentos institucionais do município analisados foram o plano de contingência para infecção humana do novo coronavírus (SARS-CoV-2), decretos, notas técnicas e notícias do *site* da prefeitura municipal com referência ao processo de trabalho da APS e aos profissionais e/ou usuários com hipertensão e diabetes, perfazendo 28 documentos (Quadro 1).

**Quadro 1:** Documentos institucionais relacionados à atenção primária à saúde desenvolvida em um município do Nordeste, 2023.

	Documento	Conteúdo relacionado à atenção primária à saúde
D1	Plano de contingência para infecção humana do novo coronavírus (Sars-CoV-2) — 03/2020.	Apresenta os fluxogramas para atendimento dos casos suspeitos e confirmados nos diversos níveis de atenção. APS: garantir atendimento e testagem nas unidades ou encaminhamento para unidades coletadoras.
D2	Decreto Municipal de 16/03/2020	Suspensão de férias e licenças dos servidores lotados na Secretaria Municipal de Saúde; Execução de atividades remotas para servidores municipais com mais de 65 anos.
D3	Decreto Municipal de 18/03/2020	Aumento da quantidade de medicamentos dispensados e da prorrogação do prazo de validade das receitas.
D4	Decreto Municipal de 18/03/2020	Possibilidade de terceiros retirarem medicamentos para evitar o deslocamento de idosos aos postos de saúde; Antecipação da campanha de vacinação da gripe; Estabelecimento de trabalho remoto para servidores municipais com mais de 60 anos, gestantes, com doenças crônicas e respiratórias e que utilizem medicamentos imunossupressores.
D5	Decreto Municipal de 19/03/2020	Suspensão dos serviços odontológicos eletivos.
D6	Nota Técnica — Novo Coronavírus n° 01/2020, de 19/03/2020	Estabelece orientações para organizar a atenção primária à saúde no enfrentamento do novo coronavírus (Covid-19) no município. Estabelece fluxo para atendimento do sintomático respiratório na atenção primária, com acolhimento, testagem, notificação, manejo clínico, encaminhamento, uso de EPI, medidas de prevenção e controle individuais e comunitárias, busca ativa e suspensão de atividades em grupo. Estabelece o papel do ACS, da equipe NASF e da equipe de saúde bucal na execução do fluxo de acolhimento a usuários com sintomas gripais.
D7	Nota Técnica — Novo Coronavírus n° 02/2020, de 25/03/2020	Atualiza as orientações para organizar a atenção primária à saúde no enfrentamento do novo coronavírus (Covid-19) no município.
D8	Nota Técnica — Novo Coronavírus n° 03/2020, de 08/04/2020	Atualiza as orientações sobre o monitoramento clínico a cada 24 h para usuários idosos ou com comorbidades de risco que se apresentem como casos suspeitos.
D9	Nota Técnica — Novo Coronavírus — n° 05/2020, de 09/04/2020	Estabelece orientações específicas sobre afastamento e retorno às atividades de profissionais de saúde.
D10	Decreto de 20 de abril de 2020	Afastamento do trabalho "in loco" de funcionários que tenham 60 ou mais anos de idade, que tenham histórico de doenças respiratórias e doenças crônicas, gestantes ou que utilizam medicamentos imunossupressores.
D11	Decreto de 20 de abril de 2020 (republicado no DOM Edição Extra de 24/04/2020)	Obrigatoriedade do uso de máscaras nos ambientes de trabalho para todos os estabelecimentos que não estejam com as atividades suspensas, inclusive repartições municipais. Esses estabelecimentos deverão fornecer as máscaras para os respectivos colaboradores.
D12	Nota Técnica DAS/APS — Novo Coronavírus n° 09/2020, de 23/06/2020	Orientações para reorganização do processo de trabalho da APS no enfrentamento do novo coronavírus no município. Estabelece os fluxos de acolhimento à demanda espontânea 1 e 2, com direcionamento das categorias profissionais para cada fluxo.
D13	Reportagem em *site* da prefeitura (30/09/2020)	Postos de saúde já realizam testes para detecção da Covid-19.
D14	Reportagem em *site* da prefeitura (30/01/2021)	Lista de unidades de saúde da família com pontos de vacinação contra Covid-19.
D15	Reportagem em *site* da prefeitura (04/02/2021)	Lista de unidades de saúde da família com pontos de vacinação contra Covid-19.
D16	Reportagem em *site* da prefeitura (20/03/2021)	Prefeitura amplia postos de saúde com testes para detecção da Covid-19.
D17	Reportagem em *site* da prefeitura (10/04/2021)	Lista de unidades de saúde da família com pontos de vacinação contra Covid-19.
D18	Reportagem em *site* da prefeitura (14/04/2021)	Lista de unidades de saúde da família com pontos de vacinação contra Covid-19.
D19	Reportagem em *site* da prefeitura (03/05/2021)	Lista de unidades de saúde da família com pontos de vacinação contra Covid-19.
D20	Reportagem em *site* da prefeitura (03/10/2021)	Lista de unidades de saúde da família com pontos de vacinação contra Covid-19.
D21	Reportagem em *site* da prefeitura (09/12/2021)	Lista de unidades de saúde da família com pontos de vacinação contra Covid-19.
D22	Reportagem em *site* da prefeitura (18/01/2022)	Lista de unidades de saúde da família com pontos de vacinação contra Covid-19.
D23	Reportagem em *site* da prefeitura (21/01/2022)	Lista de unidades de saúde da família com pontos de vacinação contra Covid-19.
D24	Reportagem em *site* da prefeitura (25/02/2022)	Lista de unidades de saúde da família com pontos de vacinação contra Covid-19.
D25	Reportagem em *site* da prefeitura (30/04/2022)	Lista de unidades de saúde da família com pontos de vacinação contra Covid-19.
D26	Reportagem em *site* da prefeitura (02/05/2022)	Lista de unidades de saúde da família com pontos de vacinação contra Covid-19.
D27	Reportagem em *site* da prefeitura (09/06/2022)	Lista de unidades de saúde da família com pontos de vacinação contra Covid-19.
D28	Reportagem em *site* da prefeitura (26/06/2022)	Lista de unidades de saúde da família com pontos de vacinação contra Covid-19.

D: documento; APS: atenção primária à saúde; EPI: equipamento de proteção individual; ACS: agente comunitário de saúde; NASF: Núcleo de Apoio à Saúde da Família; DOM: Diário Oficial do Município;

DAS: Diretoria de Atenção à Saúde.

Fonte: *Site* da Secretaria Municipal de Saúde.

Para as entrevistas, com roteiro semiestruturado, utilizou-se um gravador para registrar o áudio, que posteriormente foi transcrito. Todas as entrevistas foram obtidas de forma presencial, com um profissional de nível médio, um técnico de enfermagem e um profissional de nível superior, ou seja, três profissionais de cada uma das doze unidades de saúde da família (USFs) da região administrativa, no momento da produção dos dados.

O critério de seleção utilizado foi: profissionais das equipes que atuaram na APS nas 12 USFs, no período da pandemia da Covid-19 (março de 2020 a junho de 2022). Em uma unidade, não houve a participação do profissional técnico de enfermagem.

O presente estudo teve como referencial teórico os elementos que compõem o processo de trabalho de Mendes-Gonçalves^17^. A teoria do processo de trabalho considera como momentos essenciais a atividade adequada a um fim, ou seja, o próprio trabalho, o objeto de trabalho e os instrumentos ou meios do trabalho^17^. O objeto analisado foram as práticas de saúde ofertadas pelos profissionais da APS para o cuidado integral e longitudinal dos usuários com hipertensão e diabetes e o modo de se organizar para prestar a assistência necessária a essa população.

O resultado do processo de trabalho envolve as necessidades (carecimento) de saúde dos usuários com hipertensão e diabetes e as práticas (objeto) ofertadas pelos profissionais (agentes) da APS para que os usuários alcancem respostas (produto) para suas demandas de saúde, sejam elas biológicas, físicas, mentais, espirituais ou ambientais. As práticas de saúde consideram as dimensões objetivas do trabalho e, nos serviços de saúde, possibilitam uma reflexão sobre os modelos de atenção por meio da análise das relações sociais, que transversalizam tais práticas, e das representações sociais dos agentes sobre a situação de saúde e sua inserção nas instituições^18,19^.

O profissional da saúde, além de um "técnico de necessidades sociais de saúde", é também um "gerente de processos coletivos de trabalho em saúde"^18,19^. Os agentes considerados para este estudo foram enfermeiros(as), dentistas, técnicos(as) de enfermagem e agentes comunitários de saúde (ACS). Foram analisados nas USFs os instrumentos utilizados pelos agentes, considerando a disponibilidade e a adequação de equipamentos, a estrutura física e os saberes imateriais, como formações, vivências e experiências dos profissionais. Os instrumentos de trabalho apresentam a capacidade de sintetizar as qualidades do objeto e o projeto para chegar ao efeito desejado^17^.

As entrevistas foram anonimizadas, transcritas e revisadas com auxílio do *software* NVIVO 11. O material empírico produzido foi organizado e analisado no mesmo *software*. Todo o material coletado foi organizado, codificado e analisado conforme as categorias do referencial do processo de trabalho em saúde^17^: agentes, objeto e instrumentos. Foi adotada a triangulação de dados como estratégia metodológica, articulando múltiplas fontes e técnicas de coleta^20^. A sistematização e a análise dos dados seguiram o plano de análise (Quadro 2).

**Quadro 2:** Matriz de análise de categoria e elementos conceituais que compõem o processo de trabalho dos profissionais das equipes de saúde da família em um município da região Nordeste, 2023.

Categoria de análise	Elementos conceituais	Perguntas e observações no roteiro	Fontes de verificação
Processo de trabalho	Agentes	Qual a sua profissão? Qual sua maior titulação? Data de nascimento? Sexo? Vínculo com o município. Data do vínculo? Cor da pele (autorreferida)? Além do trabalho na unidade de saúde, você tem outra ocupação? Qual a renda familiar aproximada (em salários-mínimos)?	Entrevista com os profissionais das unidades de saúde da família
	Objeto	Quais as ações programáticas desenvolvidas nesta unidade? Como a unidade se organizou para ofertar ações não relacionadas à Covid-19 de forma concomitante às ações voltadas à Covid-19 (explorar acolhimento Covid e não Covid e ações programáticas — como as equipes se organizaram)? O que ocorreu com o trabalho do ACS durante a pandemia? Nos documentos, foram observados os protocolos e as definições da prefeitura municipal no que se refere a condições crônicas da hipertensão e da diabetes no fluxo da rede?	Entrevista com os profissionais das unidades de saúde da família Documentos da SMS publicitados de março de 2020 a junho de 2022
	Instrumentos materiais e imateriais	Havia EPI suficiente e adequado? Cite algumas experiências anteriores ao vínculo com a SMS. Você havia realizado capacitação sobre teste rápido e autoteste de Covid-19? Você já tinha experiência em curso *online?* Foram observados nos documentos os saberes materiais — estrutura física das unidades, EPIs, *tablets*, celulares, prontuários? Foram observados, nos documentos, os saberes imateriais — cursos, treinamentos e capacitações dos profissionais, assim como experiências anteriores?	Entrevista com os profissionais das unidades de saúde da família Documentos da SMS publicitados de março de 2020 a junho de 2022

SMS: Secretaria Municipal de Saúde; ACS: agente comunitário de saúde; EPI: equipamento de proteção individual.

O estudo obteve aprovação do Comitê de Ética em Pesquisa (CEP) da Organização Mundial da Saúde, conforme pareceres ERC WHO CERC.0128A e CERC.0128B; CAAE 53844121.4.1001.5030.

## RESULTADOS E DISCUSSÃO

### Mudança no Processo de Trabalho Considerando o Novo Cenário Epidemiológico

Em março de 2020, início da pandemia da Covid-19, a SMS lançou um plano de contingência que atribuiu à APS a responsabilidade pela reorganização dos seus fluxos de atendimento nas USFs e nos territórios. As ações incluíram resposta às demandas da Covid-19 e da rotina da APS, monitoramento e orientação da população, testagem ou encaminhamento para coleta, além da promoção de medidas preventivas.

O território analisado possuía mais de 400 mil habitantes assistidos por 12 USFs e 46 equipes. De acordo com a Política Nacional de Atenção Básica, cada equipe da APS em áreas urbanas deve acompanhar até 4 mil pessoas e cada ACS, até 750^21^. O déficit de equipes, associado à alta transmissibilidade da doença, contribuiu para sobrecarga dos profissionais e priorização das demandas relacionadas à Covid-19 (Quadro 3).

**Quadro 3:** Práticas de saúde desenvolvidas pelos profissionais da atenção primária à saúde, de março de 2020 a junho de 2022, em um município do Nordeste, 2023.

Unidades de saúde	Ações de resposta à Covid-19				Atenção às condições crônicas — hipertensão e diabetes			
	Testagem	Ações de vigilância	Vacina	Evidências	Planejamento	Ações programáticas realizadas	Atividades	Evidências
USF 1	Realizava	Não realizava rastreamento; Realizava telemonitoramento; Não realizava busca ativa.	Foi ponto de vacinação contra Covid-19	"Não, rastreamento, não. A gente fazia em quem chegava aí, entendeu? A gente sugeria. Por exemplo, vinha uma pessoa, uma esposa, a gente sugeria que o marido fizesse. Se tivesse com sintomas, sugeria, entendeu? Mas não era rastreado, não, de ligar, rastrear, não." (A24) "Tinha telemonitoramento. A gente tinha algumas pessoas que ficaram trabalhando *home office*, que eram profissionais dentro do grupo de risco, e eles faziam esse telemonitoramento. Inclusive, um dos médicos que trabalhava conosco, criou um programa *pra* telemonitoramento." (A13) "Mas busca ativa do quê? Para o Covid? […], não." (A24) "A gente ficava mais aqui, na área, organizando ficha, organizando cadastro, essas coisas. Recebia mais os pacientes aqui no posto." (A1)	Não estruturado; Não sistemático; Não participativo.	Apenas atendimentos voltados para Covid-19	Apenas atendimentos voltados para Covid-19	"Suspendeu logo no início da pandemia. Logo no início, a gente ficou voltado só *pra* atendimento da Covid." (A13) "Tinham uma escala, tinha uma escala." (A24) "Tinha uma escala, tinha um planejamento de como funcionaria o fluxo, tanto que aqui, na unidade, tinha o acolhimento, hoje, que a gente chama de fluxo B, que a gente determinou os fluxos como fluxo A e B, que o A é sintomático e B é o acolhimento a pacientes que não são sintomáticos respiratórios," (A13). "Eu amo a área de saúde. Ajudar as pessoas, orientar as pessoas. Se eu puder ajudar em alguma coisa, ajudar." (A1) "Os pacientes, eu acho, são bem monitorados, bem assistidos, entendeu? A gente consegue dar um suporte geral, né? Ele não vem só para mim, para a boca, ele vem para boca, mas se eu detecto com alguma coisa alta, eu já encaminho, entendeu? Então, assim, eu acho bem interessante." (A24)
USF 2	Realizava	Não realizava rastreamento; Não realizava telemonitoramento; Não realizava busca ativa.	Foi ponto de vacinação contra Covid-19	"Muito informal o rastreamento. A identificação, sim, mas muito informal com o usuário, entende?" (A25) "A gente recebeu o *tablet* do… [programa da SMS], mas o programa não foi em frente, enfraqueceu, não houve estímulo, e isso foi se perdendo. E em meio aos testes, a gente não conseguiu executar." (A25) "A gente tentou algumas estratégias por grupos de WhatsApp e tal, mas a gente teve alguns problemas, que foi a discussão do trabalho do agente comunitário de saúde […], então, no final, a gente acabou optando pelo desejo de cada um, de cada um se sentir seguro, de fazer a opção de fazer busca ativa de usuário, porque, naquele início, as pessoas morriam e ninguém queria ficar responsável." (A25)	Não estruturado; Não sistemático; Pouco participativo — reunião com a equipe para definir a escala.	Hipertensão, diabetes e procedimentos	Consultas, ações pontuais via Whats, *app* de educação em saúde para gestante, sala de espera, renovação de receita, acolhimento da demanda espontânea sem agendamento para demanda programada	"Sim, eram voltados para os grupos de risco. A gente manteve o pré-natal, puericultura e pacientes com questões crônicas, como diabetes, hipertensos, TB, então isso a gente não interrompeu, não, a gente interrompeu aquela consulta clínica mais eletiva." (A25) "Um planejamento não existia, não, até porque poderia faltar alguém […] era sempre do dia ou na escala, quem estava naquele dia no colhimento e somava mais um, sempre tinha. Mas dizer que planejou para a semana toda ou planejou para o mês, não." (A14) "O meu motivo *pra* eu ter vindo trabalhar, porque eu já, antes de trabalhar, eu já passei por algumas dificuldades com familiares, de precisar e não ser bem acolhido, de não ser bem assistido, e como eu tive a oportunidade, aí realmente eu aproveitei *pra* tentar fazer um pouquinho a diferença. Porque mesmo que não tivesse a condição de atender, mas sempre ter condição de acolher, mesmo que o médico não pudesse atender hoje, venha amanhã que a gente dá um jeito, entendeu? Um bom acolhimento, é muito importante isso." (A2)
USF 3	Realizava (outubro de 2021)	Realizava rastreamento no momento da testagem do sintomático, pedindo a ele para comparecer na unidade; Não realizava telemonitoramento; Realizava busca ativa raramente.	Foi ponto de vacinação contra Covid-19	"Perguntava quantas pessoas tinham na casa, se estavam com sintomas e, se [alguém] estivesse com sintoma, mandava vir *pra* cá." (A15) "A programação que a gente fazia era perfeitamente tranquila. 2021 foi vacina, 2022 foi praticamente testagem e acho que, no final de 2022, é que foi melhorando essa questão do Covid, e a gente voltou a fazer essas atividades que eram específicas do PSF." (A26) "Raramente [referindo-se à busca ativa], porque a maioria das pessoas vinham por demanda espontânea mesmo. A grande maioria vinha por demanda espontânea." (A26)	Não estruturado; Não sistemático; Não participativo — escala por equipe/dia.	Hipertensão, diabetes e procedimentos	Consultas para atendimento clínico	"Hiperdia, que é atendimento de hipertenso e diabético, pré-natal, planejamento familiar, saúde da criança, o programa de tabagismo funciona aqui, coleta de preventivo, coleta de teste do pezinho, vacinação, curativo, testagem para teste de doenças sexualmente transmissíveis, hepatite B e C, aids e sífilis, as consultas de clínica médica, as consultas odontológicas, PSE tuberculose e hanseníase." (A26) "Da pandemia. Desde quando inaugurou a unidade, a gente só faz a visita ou cadastramento, mas a visita reduziu bastante, e atividades educativas, que é o trabalho que o ACS faz, né? Promover prevenção, promoção e proteção, não existe. Até hoje eu estou esperando." (A3) "É porque eu amo trabalhar com a atenção primária, é muito amor que eu tenho com a atenção primária, pela prevenção, eu gosto. Nunca trabalhei em um hospital, eu gosto da atenção primária." (A15)
USF 4	Realizava	Telemonitoramento realizava; Os outros profissionais relataram que não ocorria o telemonitoramento nem o rastreamento; Não realizava a busca ativa; Não realizava o rastreamento.	Não foi ponto de vacinação contra Covid-19	"Eu acho que era por causa do fluxo, que era muito grande. Era mais por isso [em relação ao telemonitoramento que não era realizado]." (A16)	Não estruturado; Não sistemático; Pouco participativo — reunião com os enfermeiros e a gerente para definir a escala	Hipertensão, diabetes e procedimentos	Consultas para atendimento clínico, renovação de receita, comunicação em saúde no trajeto de casa para a unidade	"Fazia-se tudo aqui no posto, mas durante o período da pandemia, muita coisa ficou suspensa, mas assim, depende do período que você está falando. Porque depois que começou a vacinar e diminuíram os casos, voltaram alguns serviços, mas fazia tudo, hipertensão, diabetes, pré-natal, puericultura, planejamento familiar, essas ações todas. Agora, ficou um tempo suspenso." (A27) "Rapaz, a gente passou aqui momentos difíceis… Então, a gente tentou, sim, fazer um planejamento, não se sentavam todos, porque como era pandemia, não podia ficar todo mundo junto, mas sentavam alguns, um enfermeiro de cada equipe, com a gerente e se conversava, se planejava, se escalava, mas houve, de uma maneira ou outra, houve sim. A gente se sentava *pra* se resolver, fazer escalas, entendeu?" (A4) "É questão de perfil mesmo. Desde a faculdade, eu me identifiquei com a área da saúde coletiva, desde o meu primeiro trabalho, eu já queria trabalhar na atenção básica, em PSF, mas eu já tive experiências em gestão, experiências em atenção especializada, e eu gosto da atenção básica, porque você não fica focado unicamente em tratar a doença, mas em prevenir, em promover saúde. E isso é uma coisa que encanta na atenção básica, você poder fazer as duas coisas, você poder cuidar, mas também promover saúde." (A27)
USF 5	Não realizava	Realizava busca; Realizava rastreamento no momento da testagem do sintomático, solicitando para comparecer na unidade; Telemonitoramento realizava.	Foi ponto de vacinação contra Covid-19	"A busca ativa, às vezes, alguns profissionais e os agentes de saúde. Tinha um…, eles tinham um impresso de um tempo até, para os casos, né, positivos. Tinham impresso de acompanhamento […]." (A28) "Eu acho que só em alguns casos específicos [referindo-se à busca ativa]." (A5) "O profissional que atendia, normalmente identificava, né, o paciente que precisava do monitoramento. E aí o próprio profissional fazia um monitoramento através de contato telefônico ou pelo agente de saúde, ou o agente de saúde ia *na* casa do paciente para fazer o monitoramento." (A28)	Não estruturado; Não sistemático; Pouco participativo — reuniões de equipe para definir a escala.	Hipertensão e diabetes	Consultas reduzidas e sala de espera sobre temáticas da Covid-19	"A gente reduziu, né, reduziu o número de consultas. E aí tinha a questão da escala, né? Dependia também do dia, né? Dependendo da escala, tinham alguns profissionais voltados para o atendimento dos pacientes sintomáticos respiratórios e outros estavam fazendo os atendimentos mesmo de rotina." (A28) "É porque, na verdade, não tem mais hipertensão e diabetes como era antes, que tinha o hiperdia. Na verdade, é consulta com clínico mesmo, e eles atendem também hipertensos e diabéticos." (A28) "A gente sempre discutia em reunião de equipe." (A5) "É a questão do salário [risos] e, também, porque, assim, a forma […] você trabalhar assim, ser mais próximo da comunidade. As ações que são inseridas na unidade de saúde, que eu acho muito interessante esse contato direto que a gente tem com o paciente." (A17)
USF 6	Realizava (por um período de 1 mês)	Realizava busca ativa em casos específicos; Não realizava rastreamento; Telemonitoramento não era constante.	Foi ponto de vacinação contra Covid-19	"Você falou do… [programa da SMS] e eu lembrei que chegou, realmente, um…, mas chegou já no final. No final, não, mas numa época em que a pandemia não estava tão em alta. Demorou bastante para chegar, mas a gente tinha o…, às vezes, o monitoramento do paciente. Não era uma coisa constante." (A29) "A não ser quando a referência contactava a unidade e pedia pra fazer busca ativa desse paciente. Mas não era nada assim…, por exemplo, se a gente sabia que determinado paciente tinha Covid; não era nada certo, assim." (A18)	Não estruturado; Não sistemático; Pouco participativo — reunião de equipe uma única vez. A gerente fazia a escala.	Hipertensão e diabetes	Consulta — demanda espontânea	"No início, *pra* se definir como seria esse acolhimento, teve uma reunião mas, depois, seguia uma escala pré-organizada." (A18) "Dos programas de rotina, acho que quase nenhum. Provavelmente, planejamento e obstetrícia, *pra* acompanhamento de pré-natal. Acredito que só esses… [referindo-se aos programas de atenção a criança, diabetes, tuberculose, saúde bucal] é, foi interrompido." (A29) "Tinha a orientação, como deveria ser orientado o usuário quando ele chegasse, por exemplo, paciente de hiperdia, quando ele chegasse, que precisasse de uma troca de receita, se fosse um paciente que realmente tivesse com aquela receita vencida, era atendido no acolhimento, *pra* passar pra o médico da equipe, *pra* poder o médico da equipe renovar essa receita, nem sempre precisando de uma consulta. Eu acho que interferiu muito na questão dos atendimentos *pra* esses pacientes." (A18) "Ah, eu sou apaixonada pela área de saúde." (A6)
USF 7	Não testava	Busca ativa realizava; Realizava telemonitoramento; Realizava rastreamento no momento do telemonitoramento.	Foi ponto de vacinação contra Covid-19	"Pronto, como teve a determinação que os ACS só teriam que visitar os pacientes em casos de prioridade mesmo. Eles não tinham que fazer aquela visita periódica. Aí ficou bem restrito mesmo, só se soubesse mesmo que tinha um caso ali suspeito." (A30) "Existia na questão dos pacientes sintomáticos respiratórios. Mesmo antes do TQT, da testagem, nós fazíamos. A cada 48 horas, a gente ligava *pra* o paciente. No início, eram 14 dias de isolamento. Nesse período de 14 dias, a cada 48 horas, a gente ligava *pra* o paciente *pra* saber como é que estava, se alguém, por exemplo, estava com sintoma na casa, se teve uma piora, como é que o paciente estava durante os 14 dias do isolamento." (A19)_ "A mesma equipe. Geralmente, os contatos que eram rastreados eram, tipo assim, por exemplo, a gente ligava *pra* um paciente e o paciente dizia que a filha apresentou sintomas, aí a gente fazia o rastreio dessa filha, né, e explicava, né, *pra* poder vir *na* unidade *pra* gente avaliar." (A19)	Não estruturado; Sistemático; Participativo — reunião com todos os profissionais para definir a escala e a depender da necessidade.	Apenas atendimentos voltados para Covid-19	Apenas atendimentos voltados para Covid-19	"Quase nada, sendo bem honesta. Nós ficamos completamente voltados *pra* Covid. A gente suspendeu o atendimento médico, até por orientação da Secretaria de Saúde. Naquele momento da pandemia, não era *pra* atender paciente." (A19) "Fazia uma reunião por semana, o que estava dando errado, a gente corrigia, principalmente as médicas e enfermeiras." (A7) "Na verdade, na prática, não, porque, como eu te falei, nós estávamos todos voltados *pra* Covid, então acabou que os outros programas foi ficando, realmente, de lado." (A19) "Quando eu cheguei, eu me apaixonei. No início, eu achava chato, mas eu comecei a ver a questão voltada à prevenção, à educação, e aí eu me apaixonei." (A19) "Porque foi a área que eu mais me identifiquei mesmo." (A30)
USF 8	Não realizava	Busca ativa realizava- por demanda; Realizava telemonitoramento; Realizava rastreamento- no momento do telemonitoramento.	Foi ponto de vacinação contra Covid-19	"Tinha um sistema de monitoramento que o pessoal fazia, os enfermeiros, dentistas. Não sei agora se o médico fazia essa parte de monitorar, de ficar ligando *pra* saber se estava, sobre os sintomas, se tinha mais alguém na casa que tinha apresentado sintomas, se tinha feito o teste e dado positivo." (A20) "Sim, tanto eles como eu mesmo, eu fiz muitas visitas pelo celular, ligando para o paciente. Eu ligava para saber como é que estava, como é que não estava, fazia monitoramento." (A8) "Em alguns casos, sim, a gente pedia *pro* agente comunitário ir fazer essa busca ativa, né? Era mais difícil quando era paciente que não era…, era de área que não tinha agente. Aí é mais difícil, né? Aí a gente tentava ligar, tentava…, mas ir até a pessoa, às vezes, era difícil." (A31)	Não estruturado; Não sistemático; Pouco participativo — reuniões.	Demandas urgentes	Consultas, procedimentos e farmácia	"Pré-natal permaneceu, atendimento *pra* coisas mais urgentes… mas, por exemplo, as receitas de hipertensão e de hipoglicemia… *pra*, né, anti-hipertensivos e hipoglicemianos, eles foram prorrogados com a data sem ter um limite, né, que normalmente é de 6 meses… […] basicamente, a gente estava atendendo sintomáticos, pré-natal, pessoas com uma queixa mais urgente. Suspendeu preventivo também. Tudo que não fosse mais urgente teve que ser suspenso. Atividades educativas também foram suspensas." (A31) "Existiram alguns momentos. Mas, assim, na verdade, muito do que a gente fez foi… já foi determinado pelo próprio distrito. A gente teve reuniões, mas reuniões bem eventuais, que até tempo *pra* fazer reunião era bem complicado, assim, reunião com todo mundo. […] Mas eu lembro que muito foi determinado pelo distrito: como é, quem a gente ia atender, como que ia ser, quantos pacientes mais ou menos." (A31) "E, assim, isso me atraiu, porque cuidar da gente é o meu, eu amo, né? […] você poder mudar a vida das pessoas com relação à própria saúde dela […] cuidados bobos, né? Orientações com relação aos cuidados dela mesma, que ela, às vezes, não tem, mas quando você chega ali, você começa a cuidar daquela família, daquela pessoa, gente, muda tudo. É gratificante." (A8)
USF 9	Realizava	Não realizava telemonitoramento — falta de RH; Realizava busca ativa; Realizava rastreamento no momento da testagem do sintomático, solicitando para comparecer na unidade.	Foi ponto de vacinação contra Covid-19	"Porque aqui, no Distrito, tinha uma política…, tem ainda, né: onde se vacina para Covid, não se fazia a testagem. Então, a gente intercalava essa referência: ora era referência de vacina, ora era referência de testagem." (A32) "Tinha, quando, no momento do atendimento, a gente identificava os contatos e convocava, né." (A32) "E aí… mas, assim, nem sempre vinham. Mas a convocação…, inclusive, a gente dava um termo, que o paciente assinava, com o nome de todos os contatos, dizendo que ele que tinha compromisso de comparecer na unidade." (A32)	Não estruturado; Não sistemático; Participativo — reuniões.	Diabetes e hipertensão.	Consultas, curativos, farmácia, exames para marcar no sistema.	"Então, o agendamento era *online*, dos atendimentos que continuaram presenciais, porque tinham hipertensos que não bastava só renovar a receita, diabéticos que você precisava de um atendimento mais próximo. Agora, tinham outros que tinham tido consulta recente, exame recente, e a gente conseguia fazer teleconsulta. Ou, então, nessa primeira fase, solicitar exame a gente fazia por teleconsulta, né, já deixava todos os exames e receitas separados e, quando ele estava com o resultado, aí sim marcava uma presencial, que aí a gente fazia aquela consulta mais completa." (A32) "Então, o plano inicial, quem realizou foi eu, que a gerente me solicitou. Eu fiz um plano inicial e aí juntei a equipe. Aí tinha representante do NASF, tinha representante de agente comunitário, dentista, médico. E aí, a gente foi discutindo o que seria viável e o que deveria ser alterado. No final, a gente fez…, passou para o restante da unidade, e aí foi passando por categoria, por equipe e adaptando, também, de acordo com a demanda. Todo mundo pôde opinar." (A32) "Eu? Desemprego. Estava desempregada, achei esse aí e estou aqui." (A9)
USF 10	Realizava	Realizava telemonitoramento; Não realizava rastreamento; Não realizava busca ativa.	Foi ponto de vacinação antiCovid-19	"Esse tempo, eu não sei explicar, não. Mas eu sei que pacientes positivados, imediatamente, eles já entravam com esse telemonitoramento, essa consulta toda. Agora, por quanto tempo […]." (A21) "Não era feita, não, porque eles diziam que os pacientes não deixavam entrar em casa. Tinha aquela questão, também, de EPI, que eles tinham que estar todos paramentados, aí tinha aquela não aceitação dos pacientes." (A21). "[Em relação à busca ativa] Assim, nós temos… o certo era 750, então a gente sempre priorizava mais, assim, aqueles casos que a gente… Como a gente já tem um certo contato de anos com a comunidade, então a gente já sabe quem são as pessoas mais fragilizadas, mais expostas, então eram essas pessoas que a gente estava sempre […]." (A10) "[Em relação ao rastreamento dos contatos] Não, acho que era só dos pacientes que eram positivos." (A21)	Não estruturado; Não sistemático; Pouco participativo — escala por categoria profissional.	Não refere hipertensão e diabetes	Consultas	"Aqui na unidade, basicamente, só tinha eu de enfermeiro…, então, eu acabava ficando na testagem…, e foi feito escala entre os dentistas e comigo. Então, assim, a gente atendia de tudo, só que era por meio de escalas. Tinha dia que você estava escalado para estar participando do Covid e tinha dia que você estava com seus atendimentos." (A33) "Eu acho que esse… a vacinação funcionou. Tinha dias que funcionava e tinha dias que não. [Com] Essa parte de hanseníase e tuberculose a gente não tinha muito contato, porque quem faz é o médico. A gente não tinha muito contato, a gente tinha como saber se estava funcionando ou não […], tinham uns [referindo-se aos médicos] que ficavam na sala lá, atendendo gestante. Essa parte de tuberculose e hanseníase a gente não acompanha, não." (A21) "O dia da semana era o dia da equipe, era o dia. […], por exemplo, dia de terça-feira, era minha equipe, então era a minha equipe, e a gente tinha essa responsabilidade na terça-feira. Na quarta, era outra equipe." (A10) "Olhe, uma das razões que eu acho que é a principal é a questão da demanda…, então, a questão da demanda da atenção primária é bem maior, porque você tem uma quantidade maior de PSF, de unidade básica de saúde. Então, vim mais para atenção primária por conta disso e por parecer mais minha característica. Eu prefiro trabalhar com ações programadas, que eu posso ter o controle delas do que, por exemplo, com PA, com PA, nada é controlado, cada dia é uma dinâmica diferente, sabe, no hospital. Então, eu prefiro a atenção básica por isso." (A33)
USF 11	Realizava	Realizava telemonitoramento; Não realizava busca — falta de ACS; Não realizava rastreamento.	Foi ponto de vacinação contra Covid-19	"De quatro a cinco dias, a gente fazia busca ativa para saber se o paciente…, como é que o paciente se encontrava." (A11) "Sim. Depois do atendimento, todos os pacientes suspeitos ou confirmados eram enviados para mim, aí eu fazia esse monitoramento, telemonitoramento no período de 14 dias. E quando, assim que testava e recebia o resultado, eu encerrava, se ele já estivesse sem sintomas. Se continuasse sintomático, continuava fazendo." (A22) "Em um tempo, realizamos, mas não é algo contínuo. Sempre, vacina e testagem, a Secretaria de Saúde faz rodízio. Então, teve um tempo que a gente fez vacina; teve tempo que a gente fez testagem. E a Secretaria cria estratégias e *rodizia* isso entre as unidades." (A34)	Não estruturado; Não sistemático; Não participativo — escala feita pela gerente.	Crônicos	Consultas e renovação de receita	"Pronto, de atendimento, que você fala, né? A gente teve uma fase da pandemia [em] que a Secretaria restringiu o número de consultas […], como aqui inaugurou, em 2020, inaugurou no *boom* da pandemia. […] houve uma orientação do serviço ficar voltado apenas *pra* Covid…, então a gente fazia a avaliação desses pacientes sintomáticos. Atendíamos consultas dos outros programas só de pacientes crônicos, né, que tinham, precisavam de uma receita, que faziam algum tratamento contínuo. Mas a parte de prevenção, nesse momento, a gente parou de fazer *pra* dedicar a equipe à pandemia. Depois que isso foi controlando e começou a ter a vacina, os casos foram ficando mais brandos, a gente começou a ter mais disponibilidade de retomar, ter essa retomada das ações de prevenção da atenção primária." (A34) "Todo mundo teve que atuar, entendeu? A gerente fazia a escala do atendimento, de quem ia estar na escala do acolhimento ao sintomático respiratório e de quem estaria na coleta do teste. E todos os profissionais eram envolvidos nessa escala." (A34) "Vixe, você me pegou agora. De ter escolhido? Na verdade, eu não escolhi. […] quando eu fui me inscrever no concurso, no processo de seleção, eu escolhi para administração direta, só que 40 horas, com a intenção de trabalhar na urgência e emergência. Só que aí, quando eu consegui ingressar, me mandaram para a atenção primária. Eu não escolhi." (A22)
USF 12	Não testava	Não realizava busca ativa; Não realizava rastreamento; Telemonitoramento tentou-se fazer.	Foi ponto de vacinação contra Covid-19	"A gente tentava [em relação ao telemonitoramento], né, mas só tinha um celular *pra* unidade toda. Não deu certo." (A35) "Não, não tinha [em relação ao rastreamento de contatantes]. Só orientava, o médico orientava, se por acaso tivesse contato com alguém, pedia para ir fazer a testagem em algum lugar." (A23) [Em relação à busca ativa] É como eu falei, né, a gente tentava, mas não tinha como ter acesso exatamente aos pacientes, porque a gente só tinha um celular. E, depois, esse celular parou de funcionar. Pronto, aí é que não […]." (A35)	Não estruturado; Não sistemático; Não participativo — escala.	Hiperdia	Consultas	"[…] E depois nos atendimentos mais urgentes, que a demanda aqui era […] os pacientes estavam descompensados, assim, né […] muitos pacientes descompensados, de hiperdia, saúde mental, essas coisas assim que precisavam de um acompanhamento mais de perto, porque não tinha unidade de saúde aqui. Então, o foco foi nesse. E depois aumentaram alguns casos de pandemia, e a gente já atendia, também, né, os casos de Covid. Era sempre isso, as demandas normais, né, e as demandas de Covid." (A35) "Fez escala. Cada dia era uma equipe responsável, cada dia um médico, e um dia da semana fazia rodízio." (A35) "Menina, quando eu fiz o concurso, nem eu acreditava que ia trabalhar. No momento, mesmo, quando eu fiz, eu estava desempregada, precisando, e quando veio a notícia, meu Deus, eu nem acreditei. E hoje eu estou aqui, né? Eu gosto do que eu faço, gosto de ser agente, é isso." (A12)

USF: unidade de saúde da família; A: entrevistado; SMS: Secretaria Municipal de Saúde; ACS: agente comunitário de saúde; PSF: Programa Saúde da Família. TB: tuberculose; PSE: Programa Saúde na Escola; TQT: projeto "Expansão das estratégias de testagem, isolamento, quarentena e telemonitoramento na atenção primária à saúde em comunidades de alta vulnerabilidade social, no enfrentamento da pandemia de Covid-19, de duas capitais brasileiras"; NASF: Núcleo de Apoio à Saúde da Família; PA: pressão arterial.

Os usuários, nas USFs, eram inicialmente acolhidos pela equipe de demanda espontânea e, conforme a escuta inicial, direcionados a dois fluxos: sintomáticos respiratórios (fluxo 1) ou demais demandas, incluindo hipertensão e diabetes (fluxo 2). Os sintomáticos respiratórios tinham prioridade para atendimento médico, assim como pessoas que tiveram contato com casos suspeitos ou confirmados foram garantidos, conforme os documentos municipais (D1 e D6). A divisão dos fluxos visava reduzir o risco de contágio de usuários e profissionais^22^.

Medidas como aumento da validade de receitas de uso contínuo, dispensação prolongada de medicamentos (D3) e trabalho remoto para servidores com comorbidades foram adotadas para promover o distanciamento social. Diante de um cenário para o qual a APS não estava preparada, a gestão buscou normatizar novas práticas por meio de documentos oficiais. Uma APS estruturada nas necessidades da população e alinhada aos seus atributos mostrava-se essencial para uma resposta efetiva à Covid-19. O novo contexto, com a chegada da pandemia, trouxe para os profissionais da APS mudança brusca na sua rotina, pois foi necessário rápida adaptação para continuar prestando o cuidado aos usuários do seu território.

### Quem Foram os Agentes do Processo de Trabalho?

Os profissionais das unidades de saúde atuaram como agentes do processo de trabalho na produção de práticas de saúde voltadas para usuários com hipertensão e diabetes (Quadro 4).

**Quadro 4:** Perfil dos agentes que atuaram no Programa de Saúde da Família, em um município da Bahia, 2023.

Agentes	Posição (função)	Maior formação	Tipo de vínculo com o município	Ano de início na função atual	Idade (anos)	Sexo	Cor autorreferida	Renda familiar em SM	Outra ocupação
A1	ACS	Ensino médio	SI	SI	40 a 49	Feminino	Branca	1 a 2	Sim
A2	ACS	Ensino médio	SI	SI	50 a 59	Masculino	Negra	1 a 2	Não
A3	ACS	Superior	SI	SI	40 a 49	Feminino	Negra	1 a 2	Não
A4	ACS	Técnico	Estatutário	1998	50 a 59	Feminino	Negra	1 a 2	Não
A5	ACS	Superior	Estatutário	2003	50 a 59	Feminino	Preta	3	Não
A6	ACS	Superior	SI	2008	50 a 59	Feminino	Parda	1 a 2	Não
A7	ACS	Ensino médio e técnico ACS	Estatutário	2007	50 a 59	Feminino	Parda	4 a 5	Sim
A8	ACS	Ensino médio	Estatutário	2007	50 a 59	Feminino	Parda	1 a 2	Não
A9	ACS	Ensino médio	Estatutário	2007	>60	Feminino	Preta	1 a 2	Não
A10	ACS	Ensino médio	Estatutário	2007	50 a 59	Feminino	Negra	1 a 2	Não
A11	ACS	Ensino médio	Estatutário	2011	50 a 59	Feminino	Preta	1 a 2	Não
A12	ACS	Ensino médio	Estatutário	2007	50 a 59	Feminino	Preta	1 a 2	Não
A13	Técnico de enfermagem	Superior com especialização	SI	SI	30 a 39	Feminino	Parda ou negra	4 a 5	Sim
A14	Técnico de enfermagem	Superior com especialização	SI	SI	40 a 49	Feminino	Parda	3	Não
A15	Técnico de enfermagem	Superior com especialização	SI	SI	50 a 59	Feminino	Parda	3	Não
A16	Técnico de enfermagem	Superior com especialização	SI	SI	30 a 39	Feminino	Negra	4 a 5	Sim
A17	Técnico de enfermagem	Superior	REDA	2018	40 a 49	Feminino	Parda	3	Não
A18	Técnico de enfermagem	Técnico	Estatutário	2013	40 a 49	Feminino	Negra	3	Não
A19	Técnico de enfermagem	Superior	Estatutário	2013	40 a 49	Feminino	Parda	4 a 5	Sim
A20	Técnico de enfermagem	Superior com especialização	Estatutário	2013	40 a 49	Feminino	Parda	3	Não
A21	Técnico de enfermagem	Superior	Estatutário	2014	40 a 49	Feminino	Parda	4 a 5	Não
A22	Técnico de enfermagem	Técnico	Estatutário	2018	40 a 49	Feminino	Parda	4 a 5	Sim
A23	Técnico de enfermagem	Técnico	REDA	2019	40 a 49	Feminino	Negra	3	Não
A24	Dentista	Superior com especialização	SI	2020	50 a 59	Feminino	Branca	>10	Sim
A25	Enfermeiro	Superior com especialização	Estatutário	2003	40 a 49	Masculino	Parda	6 a 10	Sim
A26	Enfermeira	Superior com especialização	SI	SI	40 a 49	Feminino	Parda	>10	Sim
A27	Dentista	Superior com doutorado	SI	SI	40 a 49	Feminino	Parda	SI	Sim
A28	Enfermeira	Superior com especialização	REDA	SI	30 a 39	Feminino	Branca	4 a 5	Não
A29	Dentista	Superior com especialização	Estatutário	2014	30 a 39	Masculino	Parda	4 a 5	Sim
A30	Enfermeira	Superior com especialização	Estatutário	2013	40 a 49	Feminino	Parda	6 a 10	Não
A31	Enfermeira	Superior com especialização	Estatutário	2014	30 a 39	Feminino	Parda	6 a 10	Sim
A32	Enfermeira	Superior com especialização	Estatutário	2013	30 a 39	Feminino	Branca	6 a 10	Não
A33	Enfermeira	Superior com especialização	Estatutário	2012	30 a 39	Feminino	Branca	6 a 10	Não
A34	Enfermeira	Superior com mestrado	Estatutário	2015	30 a 39	Feminino	Preta	SI	Não
A35	Enfermeira	Superior	Estatutário	2013	30 a 39	Feminino	Branca	4 a 5	Não

A: entrevistado; ACS: agente comunitário de saúde; SM: salário-mínimo; SI: sem informação; REDA: vínculo temporário com o serviço público.

Foram analisadas entrevistas com 35 profissionais da APS (ACS, dentistas, enfermeiros e técnicos de enfermagem). A maioria declarou-se parda, preta ou negra (82,8%) e 17,1% branca. O sexo feminino foi predominante (91,4%). Quanto ao vínculo institucional, 60% dos profissionais possuíam vínculo estatutário, 8,5% eram contratados de forma temporária e 31,5% não o informaram. Em relação ao tempo de atuação na mesma unidade, 65,7% dos profissionais declararam estar na mesma unidade havia mais de dez anos, 5,7% até cinco anos e 28,6% não o informaram.

A faixa etária predominante foi de 40 a 49 anos (40%), seguida por 50 a 59 anos (31,4%) e 30 a 39 anos (25,7%). Apenas um profissional tinha mais de 60 anos. De acordo com o decreto municipal (D2), servidores com mais de 65 anos, com condições crônicas, gestantes ou em uso de imunossupressores foram direcionados para o trabalho remoto, especialmente por meio do telemonitoramento. Entre os 35 profissionais, apenas um foi realocado para essa função e outro para testagens externas à unidade.

### O Dever e a Covid-19: Práticas para os Usuários com Hipertensão e Diabetes na APS

Com a reorganização do processo de trabalho (D12), os profissionais, atuando na perspectiva da vigilância em saúde, deveriam monitorar remotamente os usuários com hipertensão e diabetes assistidos pelas equipes, registrando-o em prontuário. Com base nesse monitoramento, os profissionais deveriam desenvolver as ações de acompanhamento das condições de saúde, organização das renovações de receitas e orientações aos usuários estáveis, por meio de teleconsultas, consultas presenciais ou visitas domiciliares (Quadro 3).

Com o crescimento da incidência da Covid-19, o aumento por atendimentos nas USFs, o adoecimento dos profissionais e a insegurança para algumas atividades, priorizou-se o fluxo das necessidades impostas pela doença. Assim, ficou evidenciada nas entrevistas a suspensão ou a redução do número de atendimentos clínicos e do acompanhamento longitudinal dos usuários com hipertensão e diabetes (Quadro 3).

O diferencial dos profissionais da APS está no vínculo construído com os usuários e na sua inserção qualificada no território adscrito. Nesse contexto, o ACS exerce papel estratégico, especialmente no cuidado de pessoas com hipertensão e diabetes. Sua atuação permite aproximação contínua dos usuários, viabilizando práticas de cuidado que vão além do uso de medicamentos, como ações de educação em saúde voltadas ao fortalecimento da autonomia e do autocuidado, fundamentais diante das restrições impostas pela pandemia.

No entanto, esse potencial da APS foi comprometido por mudanças estruturais, seja no redirecionamento dos ACS para o trabalho interno das unidades, seja na priorização dos carecimentos relacionados à pandemia. Observou-se neste território que o crescimento no número de USFs e de equipes não foi proporcional ao número de ACS. Pelo contrário, houve redução no quantitativo desses profissionais nas equipes e redirecionamento de suas funções para outras unidades. Esse descompasso fragilizou o principal diferencial da APS, limitando a capilaridade das ações de cuidado contínuo, que são essenciais para controlar as condições crônicas de hipertensão e diabetes.

Para além desse acompanhamento contínuo e longitudinal, existiram práticas de saúde que, naquele momento da pandemia, não foram enfatizadas pelos profissionais, como ações integrativas e complementares, autocuidado apoiado e monitoramento por meio virtual ou mesmo das visitas do ACS (Quadro 3).

A falta de acompanhamento das condições clínicas dos usuários com as condições crônicas de hipertensão e diabetes pode ter agravado a saúde do usuário no decorrer do tempo. Recompor as equipes de APS é um desafio atual basilar para o acompanhamento longitudinal dos usuários com hipertensão e diabetes, assim como a estratégia da estratificação de risco desses usuários e o planejamento das ações facilitam a continuidade dos cuidados em tempos de trans e pós-pandemia^7^.

Foi evidenciado nas entrevistas que as práticas do cuidado aos usuários com hipertensão e diabetes se baseou na demanda espontânea às unidades (Quadro 3). A oferta organizada, pautada na estratificação de risco e no monitoramento dos usuários, não foi apontada nas falas dos profissionais, também não houve relatos de práticas de promoção da saúde e prevenção de agravos referentes à hipertensão e à diabetes (Quadro 3).

Ademais, não foi encontrado padrão de oferta das práticas de saúde aos usuários com hipertensão e diabetes relativas ao tempo de vínculo e atuação na mesma função pelos agentes sociais participantes do estudo (Quadro 5).

**Quadro 5:** Relação de agentes, instrumentos e produtos do processo de trabalho dos profissionais das equipes de saúde da família de um município da Bahia, 2023.

USF	Posição (função)	Agentes	Maior formação	Tipo de vínculo com o município	Tempo de vínculo atual	Outra ocupação	Implantação da primeira equipe CNES	Relação EqSF:EqSB CNES	Ofertou práticas para DM/HAS
1	ACS	A1	Ensino médio	SI	SI	Sim	2018	4:4	Não, apenas Covid-19
	Téc. de enfermagem	A13	Superior com especialização	SI	SI	Sim			
	Dentista	A24	Superior com especialização	SI	2020	Sim			
2	ACS	A2	Segundo grau completo	SI	SI	Não	2013	4:4	Sim, demanda espontânea
	Téc. de enfermagem	A14	Superior com especialização	SI	SI	Não			
	Enfermeiro	A25	Superior com especialização	Estatutário	2003	Sim			
3	ACS	A3	Superior	SI	SI	Não	2020	4:4	Sim, mas reduzida
	Téc. de enfermagem	A15	Superior com especialização	SI	SI	Não			
	Enfermeira	A26	Superior com especialização	SI	SI	Sim			
4	ACS	A4	Técnico	Estatutário	1998	Não	2011	4:4	Suspendeu no início
	Téc. de enfermagem	A16	Superior com especialização	SI	SI	Sim			
	Dentista	A27	Superior com doutorado	SI	SI	Sim			
5	ACS	A5	Superior	Estatutário	2003	Não	2015	2 :2	Sim, mas reduzida
	Téc. de enfermagem	A17	Superior.	REDA	2018	Não			
	Enfermeira	A28	Superior com especialização	REDA	SI	Não			
6	ACS	A6	Superior	SI	2008	Não	2005	4:1	Suspendeu no início
	Téc. de enfermagem	A18	Técnico	Estatutário	2013	Não			
	Dentista	A29	Superior com especialização	Estatutário	2014	Sim			
7	ACS	A7	Ensino médio e técnico ACS	Estatutário	2007	Sim	2013	4:4	Não, apenas Covid-19
	Téc. de enfermagem	A19	Superior	Estatutário	2013	Sim			
	Enfermeira	A30	Superior com especialização	Estatutário	2013	Não			
8	ACS	A8	Ensino médio	Estatutário	2007	Não	2006	3:3	Sim, com hora marcada, apenas demandas urgentes
	Téc. de enfermagem	A20	Superior com especialização	Estatutário	2013	Não			
	Enfermeira	A31	Superior com especialização	Estatutário	2014	Sim			
9	ACS	A9	Ensino médio	Estatutário	2007	Não	2004	4:4	Sim
	Enfermeira	A32	Superior com especialização	Estatutário	2013	Não			
10	ACS	A10	Ensino médio	Estatutário	2007	Não	2014	5:5	Não, apenas Covid-19
	Téc. de enfermagem	A21	Superior	Estatutário	2014	Não			
	Enfermeira	A33	Superior com especialização	Estatutário	2012	Não			
11	ACS	A11	Ensino médio	Estatutário	2011	Não	2020	4:4	Sim
	Téc. de enfermagem	A22	Técnico	Estatutário	2018	Sim			
	Enfermeira	A34	Superior com mestrado	Estatutário	2015	Não			
12	ACS	A12	Ensino médio	Estatutário	2007	Não	2020	4:4	Sim
	Téc. de enfermagem	A23	Técnico	REDA	2019	Não			
	Enfermeira	A35	Superior	Estatutário	2013	Não			

USF: unidade de saúde da família; A: entrevistado; ACS: agente comunitário de saúde; SI: sem informação; REDA: vínculo temporário com o serviço público; EqSF: equipe de saúde da família; EqSB: equipe de saúde bucal; CNES: Cadastro Nacional de Estabelecimentos de Saúde; DM: diabetes *mellitus*; HAS: hipertensão arterial sistêmica.

O processo de trabalho dos profissionais da APS no cuidado oferecido aos usuários com hipertensão e diabetes compreende um conjunto de práticas que extrapola as consultas clínicas por médicos e enfermeiros. Trata-se de um acompanhamento ampliado, que inclui ações voltadas para promover a adesão terapêutica, estimular a prática regular de atividade física, incentivar a alimentação saudável e o manejo do estresse, além de enfrentar dificuldades psicológicas, intensificadas pelas restrições da pandemia. Essas práticas contribuem para fortalecer o autocuidado e o autoconhecimento, favorecendo a construção de melhor qualidade de vida e autonomia dos usuários na condução de sua condição crônica.

### Os Limites e os Desafios do Novo Cenário na APS: Discrepâncias entre o Almejado e o Realizado

Ocorreu, no Brasil, aumento da incidência de usuários com hipertensão e diabetes durante a pandemia, agravando o cenário das condições crônicas, constituindo-se na chamada quarta onda da pandemia, que poderá trazer agudizações durante muitos anos a seguir^23^. Assim, o objeto do processo de trabalho dos profissionais da APS foi mais direcionado para atender às demandas da Covid-19, deixando para um segundo plano as necessidades de saúde que coexistiam no território.

No tocante ao planejamento para ações nas unidades de saúde, verificou-se um momento não programático na sua maioria, tendo as ações voltadas para a construção de escala, na qual a equipe era dividida entre as "ações Covid" e "não Covid", e também de forma não sistemática, pois ocorriam de acordo com a rotina da unidade e as demandas que emergiam, com pouca ou nenhuma participação dos profissionais da equipe para discutir as melhores estratégias ou condições de trabalho.

Em uma crise sanitária, ações precisam ser voltadas para identificar pacientes de alto risco, assim como construir um plano que organize e direcione as práticas de saúde para os usuários com condições crônicas^7^. O planejamento, na gestão em saúde, é uma forma para desenhar, organizar e acompanhar proposições, a fim de operacionalizar as decisões institucionais^24^. Desse modo, é possível verificar um planejamento estruturado e sistemático nas ações no nível da SMS, porém este não foi evidenciado nas narrativas dos profissionais em relação ao planejamento e à prática nas USFs (Quadro 3).

O "fica em casa" da pandemia levou ao afastamento dos usuários das USFs e à descontinuidade do cuidado longitudinal presencial para aqueles com condições crônicas, porém novas tecnologias foram inseridas no cenário pandêmico, possibilitando novas formas de acesso^11,16^. Para atender à rápida mudança no processo de trabalho na APS, foram necessários instrumentos que subsidiassem as práticas, preparação dos profissionais e, acima de tudo, financiamento que sustentasse essas alterações^9^.

Nessa perspectiva, a análise dos instrumentos que seriam utilizados pelos profissionais como meios de transformação para o produto final mostrou discordância sobre as capacitações dos agentes, já que alguns relataram treinamento sobre a Covid e outros referiram que o conhecimento se deu por conta própria, por meio das informações veiculadas nas mídias e repassadas pelas gerências das unidades. Outra lacuna foi a disponibilização de equipamentos de proteção individual, a exemplo de máscaras do tipo N95 (Quadro 3).

Os documentos institucionais referiam-se às novas ferramentas tecnológicas como estratégias para minimizar os riscos aos profissionais e evitar a desassistência aos usuários, principalmente dos grupos estratégicos, que incluíam usuários com condições crônicas de saúde. Entretanto, a tentativa da SMS de ampliar o acesso aos usuários na APS não foi utilizada de forma equânime nas 12 unidades da região administrativa. As práticas de cuidado dos usuários com hipertensão e diabetes na APS demandam mudanças tanto de profissionais e gestores preparados para atuar no cuidado e abertos a romper com o modelo hospitalocêntrico quanto de políticas públicas que subsidiem um novo modelo de atenção, além de usuários empoderados e corresponsáveis por seu processo de saúde^6^.

Em um cenário em que a APS contava com áreas descobertas, ACS voltados para o trabalho burocrático dentro das unidades, redução de consultas presenciais ou virtuais, prescrições com validade prorrogada sem verificar o controle dos parâmetros e falta ou insuficiência de equipamentos para monitorar essa subpopulação, além do próprio medo dos profissionais de se contaminarem, as limitações no processo de trabalho foram veementemente evidenciadas nas entrevistas dos profissionais da APS (Quadro 3).

As práticas de saúde ofertadas aos usuários com hipertensão e diabetes foram baseadas na demanda espontânea, principalmente para renovação das receitas. As práticas de prevenção de doenças e promoção de saúde não constam das evidências do presente estudo. Os agentes desse processo precisam ter condições para conhecer seus usuários e o território no qual estão inseridos, para isso, faz-se necessário ampliar as equipes de saúde com ACS e o quantitativo de profissionais, além de estruturar o processo de trabalho. Os agentes sociais são movidos por uma tensão de vontade para mudar o produto final do seu processo de trabalho.

O prejuízo no acompanhamento e no acesso à APS no tocante aos usuários com hipertensão e diabetes, na pandemia, também foi identificado em outras localidades, o que impôs aos trabalhadores a readequação do seu processo de trabalho^25,26^. Evidenciaram-se práticas de saúde pautadas na demanda espontânea, com fragilidades no monitoramento dos usuários assistidos pelas equipes e enfoque na renovação de receitas, semelhantemente a experiências em demais territórios do país^4,25,26^.

A pandemia da Covid-19 mostrou uma mudança no produto final do processo de trabalho, que foi mais direcionado aos efeitos da pandemia do que relacionado, de forma abrangente, às melhorias na saúde da população contempladas pelo olhar da APS.

## CONCLUSÃO

Diante da nova realidade imposta pela pandemia da Covid-19, fica evidenciado que esse período mostrou alteração nas práticas de saúde ofertadas pelos profissionais da APS. Naquele momento, foi preciso, para esse agente, reinventar-se, mesmo com pouca habilidade/recurso para a nova realidade e com alta demanda pelas novas necessidades de saúde impostas pelo cenário^4,25^.

Houve distanciamento real dos usuários com hipertensão e diabetes das unidades, seja por medo da contaminação ou pelo redirecionamento da atenção dos profissionais às demandas da Covid-19, porém com o avançar da pandemia, a chegada da vacina e, consequentemente, a diminuição da incidência dos casos, os atendimentos retornaram lentamente, mas com características limitadas.

Destaca-se que as práticas de saúde oferecidas aos usuários com condições crônicas, como hipertensão e diabetes, seguem fragmentadas e precisam ser retomadas/qualificadas, porém pautadas na equidade, com planejamento, monitoramento e conhecimento do seu território, para que se possa ofertar o acesso integral e resolutivo a essa população, que continua "isolada", mesmo com o fim da pandemia.

## Data Availability

Os dados da pesquisa não incluídos no artigo não estão disponíveis.
